# Photic Cycle Shorter Than or Equal to Endogenous *tau* Postpones Diet‐Induced Obesity in Mice and Shows a Robust Aftereffect

**DOI:** 10.1096/fj.202502574RR

**Published:** 2026-09-01

**Authors:** Anat Neumann, Roee Gutman

**Affiliations:** ^1^ Department of Animal Sciences, Faculty of Sciences and Technology Tel‐Hai University of Kiryat Shmona in the Galilee Upper Galilee Israel

**Keywords:** circadian disruption, circadian rhythms; aftereffect, locomotor activity, obesity

## Abstract

High‐fat diet (HFD)‐induced obesity (DIO) is preceded by disruptions in endogenous circadian rhythmicity, including lengthening of its period (*tau*). We previously demonstrated that housing mice under a light–dark cycle (T‐cycle) oscillating at their endogenous *tau* of 23.7 h prevents the DIO found under the 24‐h T‐cycle, suggesting that DIO is also secondary to disruptions caused by entrainment to non‐*tau*‐matching T‐cycles. Here, we aimed to test this hypothesis by comparing energy homeostasis of low‐fat diet (LFD) and HFD‐fed female mice housed under four regimes: constant darkness (DD), a T‐cycle oscillating at the *tau* of age‐matched LFD‐fed mice, a non‐*tau*‐matching 24‐h T‐cycle, or a T‐cycle shorter than *tau* by the age‐appropriate *tau*‐24‐h deviation (∆*tau*). In LFD‐fed mice, energy homeostasis was unaffected by photic regime. In contrast, while HFD induced DIO across all regimes, onset occurred at similar times under DD and the 24‐h T‐cycle but was similarly delayed by 8 weeks under both the *tau*‐like and ∆*tau* T‐cycles. Notably, DIO onset under DD was preceded by the prevention of *tau* shortening, a characteristic of LFD‐fed mice. Delayed DIO was not explained by reduced energy intake; instead, it was associated with a preserved locomotor activity level, suggesting higher energy expenditure, and was followed by a stronger (compared to 24‐h T‐cycle) *tau*‐shortening aftereffect. In conclusion, these findings identify HFD‐induced *tau* lengthening as a mechanism promoting early DIO under the conventional 24‐h T‐cycle. Importantly, they suggest that *tau*‐shortening pharmacological or nutritional interventions may postpone DIO in susceptible individuals and settings, even during ad‐libitum HFD feeding.

## Graphical abstract

Feeding female mice a high‐fat diet (HFD) lengthens endogenous circadian rhythmicity (*tau*) prior to HFD‐induced obesity (DIO), characterized by increased energy intake and reduced locomotor activity (A). While this DIO also occurs under a slower‐than‐*tau* 24 h T‐cycle, aligning T‐cycles to oscillate faster than the HFD‐lengthened *tau* postpones DIO—a delay energetically explained by preserved locomotor activity levels (B). This postponement likely stems from the T‐cycle‐induced *tau*‐shortening aftereffect (C), which may reinforce circadian and metabolic homeostasis against obesogenic challenges.

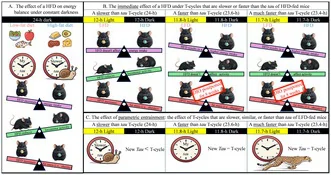

## 1 Introduction

Most physiological parameters exhibit sustained circadian rhythms that oscillate under constant darkness (DD) with an age‐dependent period length (*tau*) [1, 2]. These rhythms are generated by the suprachiasmatic nucleus (SCN)‐located master clock that perceives light–dark cycle timing signals, thereby entraining the master clock and, in turn, peripheral clocks [3]. Entrainment is carried out using the non‐parametric [4] and the parametric modes of entrainment [5]. The parametric mode of entrainment is characterized by changes in *tau* length in accordance with the length of the entraining light–dark cycle (T‐cycle), referred to as an “aftereffect” [5]. Circadian disruption of endogenous rhythms precedes and predisposes mice to metabolic syndrome (MS) and obesity when fed either a low‐fat diet (LFD) or a high‐fat diet (HFD), offering circadian disruption as a mechanistic explanation for obesity [6]. These circadian disruptions and the preceding HFD‐induced obesity (DIO) are prevented by time‐restricted feeding, which consolidates circadian rhythms, suggesting that DIO is secondary to HFD‐induced circadian disruptions [7, 8].

These circadian disruptions were also found to be related to a T–cycle–*tau* deviation, as we and others have found that wild‐type (WT) mice with a *tau* closer to 24‐h showed lower susceptibility to DIO and longer lifespans under a 24‐h T‐cycle [1, 9, 10, 11, 12]. Moreover, mutated mice and hamsters housed under their *tau*‐resembling T‐cycles of 22 or 20 h, respectively, showed reduced DIO levels (in mice), and lower metabolic syndrome and longer lifespan (in hamsters), than under the 24‐h T‐cycle [13, 14, 15, 16]. Notably, these phenotypes were absent in WT and arrhythmic‐muted hamsters housed under a 24‐h T‐cycle, and in free‐running mutated hamsters [13, 14, 15, 16]. Taken together, these results support the “circadian resonance” hypothesis [17], which argues that a chronic misalignment between *tau* and an entrainable T‐cycle leads to metabolic disruptions resulting from entrainment‐related biochemical disruptions [10, 12]. None of these studies, however, challenged the relevance of the “circadian resonance” hypothesis to “real‐world” conditions by testing whether entrainment of WT to a T‐cycle diverting from *tau* by approximately 0.3–0.5 h also results in disrupted metabolism, particularly under HFD. This slight *tau*‐T‐cycle difference better describes the “real‐world” conditions in which mice and humans are housed under the “regular” 24‐h T‐cycle, while their *tau* is about 23.7 h [18] and 24.5–25.0 h [19], respectively.

We recently addressed this challenge and showed that while housing LFD‐fed mice under their *tau*‐like 23.7‐h T‐cycle did not affect energy balance, it prevented the HFD‐induced circadian disruption and the subsequent DIO found under the 24‐h T‐cycle [2]. Hence, we suggested that DIO under the 24‐h T‐cycle is due to a “double‐hit” process [2]. The first hit is due to entrainment to a non‐*tau*‐like T‐cycle (even with a 0.3 h deviation), rendering the homeostatic system vulnerable to the second hit—the HFD‐induced circadian disruption manifested by DIO, whose rate is related to the T‐cycle‐*tau* deviation. Following this suggested model, the master clock can be strengthened by eliminating the need for entrainment, as achieved under DD or by imposing a T‐cycle Zeitgeber oscillating at a *tau*‐like length, which not only removes the need for entrainment but also further strengthens the endogenous clock. We argued that this strengthened circadian homeostasis may enable a more adequate circadian and energy homeostasis, even during ad libitum HFD feeding [2].

Notably, both Zhou et al. and our studies [2, 16] have the same flaws: 1. mice were held under a fixed *tau*‐like T‐cycle, while *tau* is age‐dependent [9, 20, 21], and 2. DIO under the *tau*‐like T‐cycle was compared only to that obtained under a longer‐than‐*tau* 24‐h T‐cycle, rather than also to that under DD and a shorter‐than‐*tau* T‐cycle (e.g., a 23.4‐h T‐cycle). The latter issue is relevant when translating results to humans, whose *tau* is longer than 24‐h [19]. Therefore, this study aimed to examine the “double‐hit” model of DIO under the “regular” 24 T‐cycle and address the flaws in our previous work. By doing so, our results may better elucidate the mechanisms underlying human DIO under the “regular” 24‐h T‐cycle, which is misaligned with their *tau*.

## 2 Materials and Methods

### 2.1 Animals, Experimental Protocol, and Diets

**FIGURE 1:**
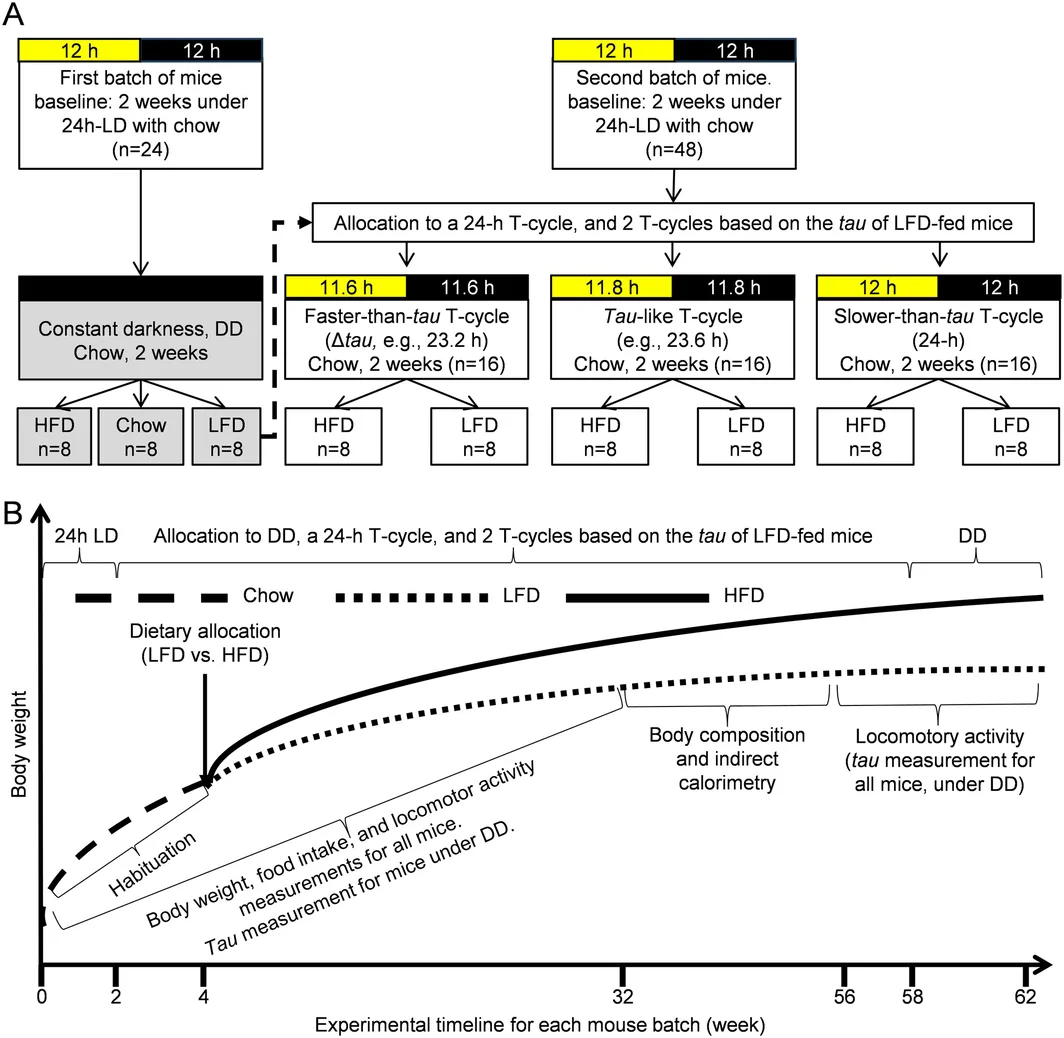
Experimental design. (A) Schematic overview of the experimental design for each batch of three‐week‐old C57BL6/J female mice (*n* = 72). Boxes indicate assignment to photic regimes, and dietary allocation to a low‐fat diet (LFD) and a high‐fat diet (HFD), for the first batch of mice (*n* = 24) and the second batch (*n* = 48), which arrived 4 weeks later. This 4‐week difference in arrival time allowed using the average age‐appropriate circadian locomotor activity period length (i.e., *tau*) of LFD‐fed mice housed under constant darkness (DD) to set and adjust the period length of the *tau*‐like T‐cycle and the Δ*tau* T‐cycle, which run faster than the *tau*‐like T‐cycle by the age‐appropriate deviation of *tau* from 24‐h. (B) Timeline of assignment to photic regimes (at 2 weeks on experiment), dietary allocation (at 4 weeks on experiment), and data collection timepoints for key variables along the study course. Data collection included recordings of continuous locomotor activity, weekly or biweekly measurements of body weight and food intake, body composition assessments, indirect calorimetry for energy expenditure, and *tau* measurements taken before the experimental endpoint to detect after‐effects.

The experiment complied with the guidelines of the Israel Committee for Animal Experimentation. Seventy‐two C57BL/6J (B6) 3‐week‐old female mice (Envigo, Israel) were ordered in two batches, 4 weeks apart (Figure 1A). We intentionally chose females, unlike most animal studies using males [22], as females are underrepresented in DIO studies, despite the higher proportion of DIO in human females [23]. Mice were single‐housed in a temperature‐controlled facility (23°C–24°C) with a symmetrical (half‐light half‐dark hours) 24‐h T‐cycle and fed ad lib on water and regular rodent chow (2018S, Envigo; 3.1 metabolizable kcal per gram). After 2 weeks of habituation and basal measurements (see below), the first batch (*n* = 24) was transferred to DD for *tau* measurement (Figure 1A). Their age‐appropriate *tau* was used to set and adjust the period length of two T‐cycles, as explained below (Figure 1A). Following 2 weeks under DD, mice were switched to one of two diets (Envigo, *n* = 8 per diet): a LFD (TD.08806, 3.6 kcal/g, 10% fat kcal), a HFD (TD.06414, 5.1 kcal/g, 60% fat kcal), or remained on chow (*n* = 8. Figure 1A). Allocation was semi‐randomized, so groups had similar means and SEM of body weight and *tau* at the time of allocation. The *tau* of LFD‐fed mice, rather than that of chow‐fed mice, determined the T‐cycle regimes for the other groups because LFD is identical to HFD except for fat content. However, because many studies used chow as the control for HFD‐induced circadian disruption [21, 24, 25], one group remained on chow for comparison (Figure 1A).

The second batch of 3‐week‐old female mice (*n* = 48) arrived 4 weeks later than the first batch, housed under a 24‐h T‐cycle for habituation and basal measurement for 2 weeks (Figure 1A). Then, they were assigned to one of three symmetrical T‐cycles: (1) an age‐appropriate *tau*‐like T‐cycle, based on the average *tau* of same‐age LFD‐fed mice under DD (*tau*‐like T‐cycle), (2) a *tau*‐mismatched “regular” 24‐h T‐cycle (24‐h T‐cycle), or (3) a *tau*‐mismatched T‐cycle shorter than *tau* by the age‐appropriate deviation of *tau* from 24‐h (Δ*tau* T‐cycle, Figure 1A). The period length of the *tau* and Δ*tau* T‐cycles was adjusted biweekly (to a one‐minute resolution per light and dark cycle, i.e., a 0.033‐h resolution) using the *tau* of LFD‐fed mice under DD. Two weeks after T‐cycle initiation, half of the mice in each T‐cycle were transferred to LFD or HFD (Figure 1A), with semi‐randomized allocation to ensure similar baseline mean and SEM of body weight, and similar *tau* across dietary groups at the time of allocation. Locomotor activity was recorded continuously during the first 32 weeks of the experiment, while body weight and daily food (i.e., energy) intake were measured biweekly under DD or weekly in other groups (Figure 1B). Intake was adjusted for metabolic mass (body weight^0.67^), following [26]. Energy balance and body composition were measured by indirect calorimetry between 32 and 56 experimental weeks, followed by transfer to DD for *tau* measurement (Figure 1B).

### 2.2 Measurement of Locomotor Activity and *Tau* Calculation

Individual locomotor activity was monitored at 6‐min intervals, from approximately 3 weeks of age, using our custom‐made infrared motion detector system [1, 2, 18, 27]. Locomotor activity is a direct output of the master clock [28]. Hence, ClockLab software (Actimetrics, USA) was used to calculate the period length of locomotor circadian activity rhythm under DD as a marker of the endogenous circadian master clock period length (*tau*), as done in our previous studies [1, 2, 9].

### 2.3 Measurement of Energy Balance by Indirect Calorimetry

**FIGURE 2:**
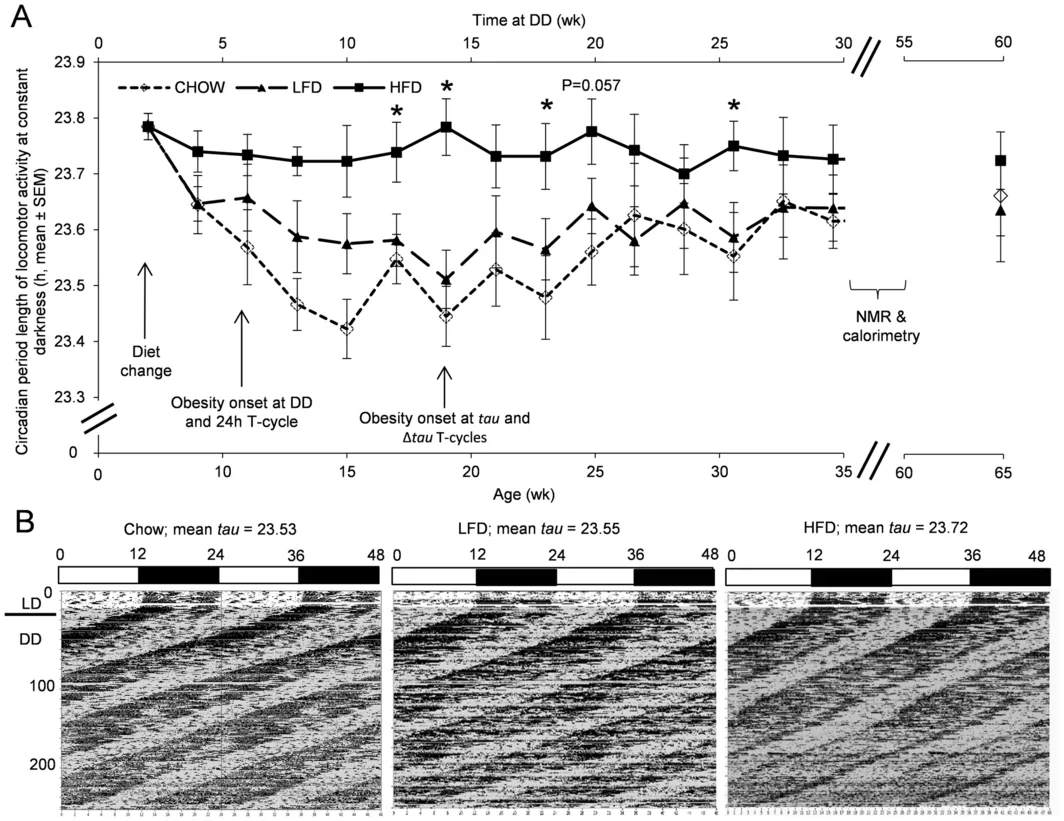
High‐fat diet prevents the age‐induced shortening of the endogenous circadian rhythm period length (*tau*). (A) Chow‐fed, five‐week‐old C57BL6/J female mice under constant darkness (DD) up to 65 weeks of age showed an age‐induced shortening in *tau*, determined by measuring the individual locomotor activity rhythm using an infrared detector. This age‐related change in *tau* was also found, to some extent, in low‐fat diet (LFD)‐fed mice. Providing a high‐fat diet (HFD) prevented age‐related changes in *tau*, resulting in significantly longer *tau* up to 32 weeks of age. *Tau* was calculated using ClockLabs' periodogram analysis toolbox for 13‐day windows. *p < 0.05 significant difference between age‐matched LFD‐ and HFD‐fed mice using the Mann–Whitney test. Arrows indicate the introduction of LFD and HFD, as well as the onset of diet‐induced obesity (DIO), under each photic regime. DIO onset was defined as the minimal time on HFD in which the body weight of a HFD‐fed mouse was significantly higher than the average body weight of age‐matched LFD‐fed mice under the same T‐cycle (using the Wilcoxon test) and differed significantly for two consecutive measurements. (B) Representative, double‐plotted actograms demonstrating locomotor activity rhythms at a 24‐h light–dark (LD) T‐cycle followed by a DD regime (gray background) and the effect of chow, LFD, and HFD diets on *tau*. White and black bars at the top of the actogram present the light and dark cycles. At the top of each actogram, the *tau* value indicates the mean tau from 21 to 29 weeks of age. The data are presented as mean ± SEM, *n* = 8 per group.

Indirect calorimetry measurements were performed from 35 weeks of age using an eight‐cage system equipped with food intake monitoring (Sable Systems, USA, Figure 1B). Since the *tau* of LFD‐fed mice under DD was found to be stable at this age (Figure 2A), no adjustments were made to *tau*‐like and Δ*tau* T‐cycles during and after calorimetry measurements. Mice were habituated to the calorimetry system for 48 h, followed by 48‐h of data collection to obtain a representative 24‐h energy intake and expenditure data. Raw data sampled for 0.5 min every 5 min and processed by ExpeData v.1.8.2 (Sable Systems). Energy expenditure (EE) per time point was calculated using Weir's equation [29], corrected for the duration of measurements, and summed to obtain a total 24‐h energy expenditure (TEE) [30]. Resting EE (REE) was defined as the lowest 0.5‐h EE during the representative 24‐h period. Non‐resting EE (NREE) was calculated as TEE − REE, which was first multiplied by 48 to extrapolate to 24‐h REE [30].

Body composition (fat mass [FM], fat‐free mass [FFM], and extracellular fluid [fluids]) was measured by Nuclear Magnetic Resonance (NMR; Minispec LF50, Bruker, Germany), at 35 weeks of age and after calorimetry (Figure 1B). Intake and expenditure rates were adjusted for body weight and composition differences (i.e., FM and FFM), as done previously by us and recommended by others [31, 32]. To do so, we defined the mouse's “metabolic mass” as its FFM plus 18% of its FM, as we found in previous work that TEE of B6 mice fed ad libitum with LFD or HFD equals 0.34 × FFM + 0.06 × FM + 5.16 (*R* ^2^ = 0.66, *p* < 0.01 [32]), that is, the covariate of FM is ca. 18% that of FFM. Total 24‐h metabolizable energy intake (TEI) was calculated as the change in food weight across the parallel 24‐h TEE measurement, accounting for dietary‐specific metabolizable caloric density. Energy balance was defined as the difference between TEI and TEE [30].

### 2.4 Statistical Analysis

Data is presented as mean ± standard error (SEM). One or two‐way repeated measures ANOVA with the false discovery rate method of Benjamini and Hochberg (i.e., FDR) post hoc analysis evaluated the effect of diet, T‐cycle, and time at DD on *tau*. The Mann–Whitney test was used to assess the impact of diet on *tau* at individual time points. Under a HFD, above 90% of the HFD versus LFD body weight difference in mice is explained by a higher fat mass, which results in a higher fat percentage [30]—that is, diet‐induced obesity (DIO). Therefore, DIO onset was defined as the minimal time on HFD in which the body weight of a HFD‐fed mouse was significantly higher for two consecutive measurements than the average body weight of age‐matched LFD‐fed mice under the same T‐cycle, using the Wilcoxon test. A one‐way ANOVA with FDR post hoc analysis test evaluated the effect of the T‐cycle on DIO onset of HFD‐fed mice (see Table S1 for a step‐by‐step description of DIO onset definition). In addition, DIO onset was also defined as the time on the HFD diet at which a significant difference in body weight between HFD‐ and LFD‐fed mice was observed, using two‐way repeated measures ANOVA with FDR post hoc analysis. A two‐way ANOVA with FDR post hoc analysis was also used to assess the effects of T‐cycle or diet on cumulative (metabolism‐mass corrected) energy intake.

**FIGURE 3:**
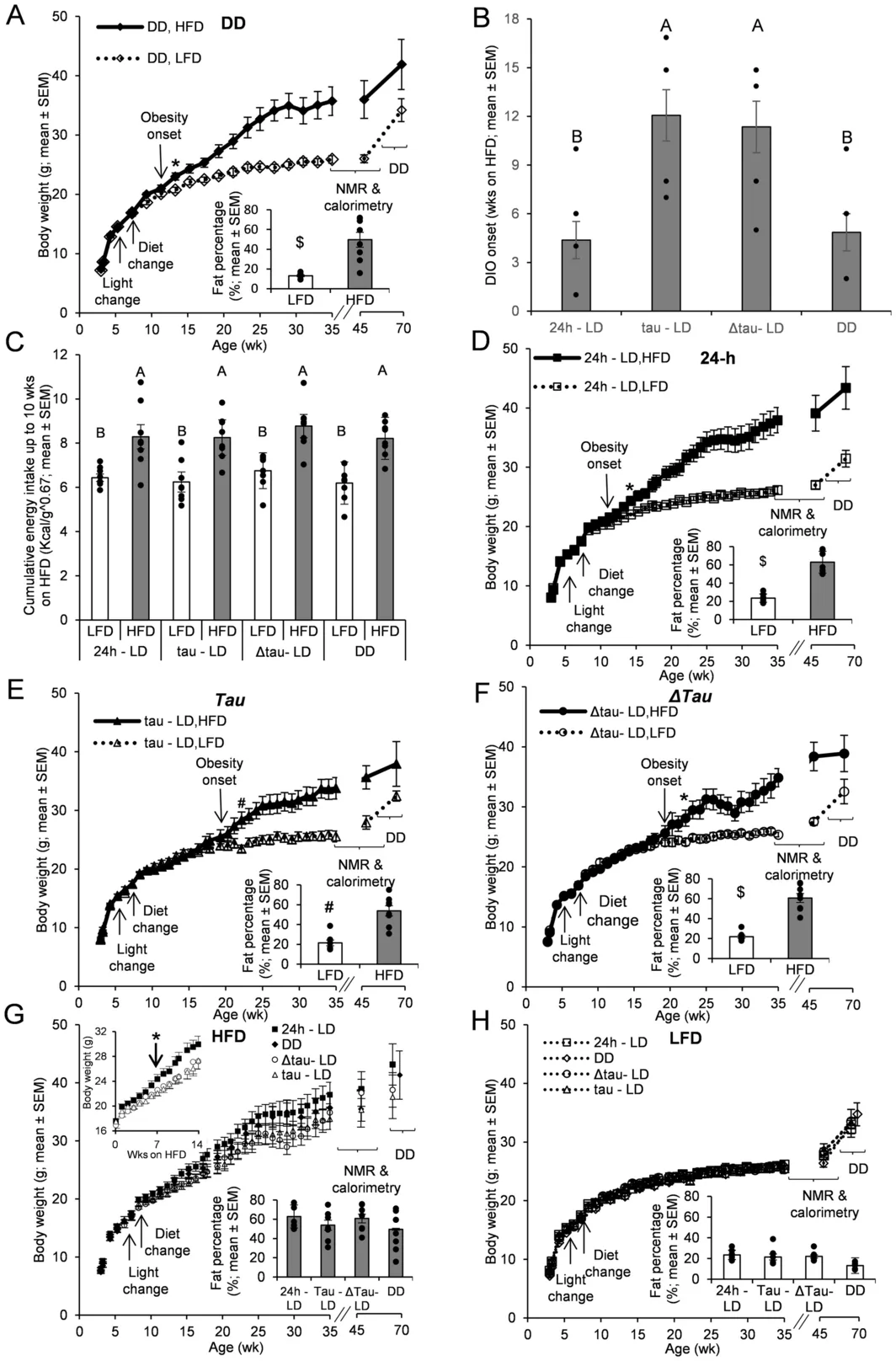
High‐fat diet‐induced obesity is postponed under a photic cycle oscillating at a period length similar to or shorter than the age‐appropriate endogenous circadian rhythm period length. (A and B, D–F) Four weeks of feeding on a high‐fat diet (HFD) was sufficient to result in HFD‐induced obesity (DIO) under constant darkness (DD) and a 24‐h T‐cycle (see B, and arrows at A and D). DIO onset, however, was postponed by ca. 8 weeks (to the 12th week on HFD) under the age‐appropriate *tau*‐like and the shorter‐than‐*tau* T‐cycles (*tau*‐LD and Δ*tau*‐LD, accordingly, see B, and arrows at E and F). The *tau*‐LD T‐cycle was a symmetrical T‐cycle with a period length that was equal to the average *tau* of the same‐age mice on a LFD and housed at DD, while the period length of the symmetrical Δ*tau* T‐cycles was set to be shorter than the *tau* T‐cycle by the age‐appropriate deviation of *tau* from 24‐h. Different letters at *B* represent significantly different DIO onset, as determined by One‐way ANOVA with FDR post hoc analysis. Fat percentage, measured by NMR before calorimetry, was higher in DIO mice (see insert in A and D–F; ^#^ *p* < 0.01; ^$^ *p* < 0.001 by Mann–Whitney test). (C) Compared to LFD‐fed mice, the cumulative (metabolic‐mass corrected) energy intake of all HFD‐fed mice was higher at the tenth week of feeding and did not differ by T‐cycle in either diet. However, only HFD‐fed mice under DD and 24‐h LD were obese at this experimental time point. Different letters represent significantly different intakes between treatments, as determined by Two‐way ANOVA with FDR post hoc analysis. (G) Between the sixth and twelfth weeks on HFD, HFD‐fed mice under *tau* and Δ*tau* T‐cycle showed lower body weight than HFD‐fed mice under 24‐h T‐cycle (insert, 24‐h vs. *tau* and Δ*tau* regimes combined). (H) T‐cycle did not affect body weight growth curves under LFD. A, D, E, F, and G (insert) *p < 0.05, ^#^ *p* < 0.01, significant difference between body weights by two‐way repeated measure ANOVA with FDR post hoc analysis. The data are presented as mean ± SEM, *n* = 8 per group.

Multiple linear regressions assessed the contribution of differences in cumulative energy intake (metabolic‐mass corrected), cumulative locomotor activity, and *tau*, on HFD versus LFD body weight difference within a T‐cycle, and among HFD‐fed mice across T‐cycles. Cumulative energy intake was calculated by multiplying daily energy intake by the number of days between measurements. Given the differences in body mass and body composition across diets and within HFD‐fed mice during calorimetry measurements (Figure 3), energy intake and expenditure rates were normalized to account for these variations in body mass and composition (see above). A two‐ or three‐way repeated measures ANOVA with FDR post hoc analysis was used to evaluate the effects of T‐cycle and diet on TEI, TEE, REE, and NREE. The Wilcoxon test was used to assess whether the net energy balance (energy intake minus energy expenditure) differed from zero. A two‐way repeated measures ANOVA with FDR post hoc analysis was used to evaluate the effect of diet and time on DD on *tau* within a T‐cycle. Thereafter, two‐way ANOVA examined the after‐effect, that is, the impact of the previous T‐cycle (and diet) on *tau*, and the Wilcoxon test assessed whether *tau* differed from the previous T‐cycle period length. All statistical analyses were conducted using SPSS version 23 (SPSS Inc., Chicago, IL, USA) or GraphPad Prism version 10.2.2 (GraphPad Software, Boston, MA, USA), with a significance level set at *p* < 0.05.

## 3 Results

### 3.1 DIO Onset Under DD Is Preceded by *Tau* Elongation

Transferring chow‐fed mice from a 24‐h light–dark cycle (T‐cycle) to constant darkness (DD) produced a free‐running locomotor activity rhythm with an age‐dependent circadian period length (*tau*; *p* < 0.001, repeated measures ANOVA, Figure 2A,B). *Tau* of chow‐fed mice shortened until ca. 15 weeks of age, rebounded and stabilized by ca. 33 weeks, remaining constant until experimental endpoint (at 65 weeks of age, 60 weeks at DD, Figure 2A,B). *Tau* was also diet‐dependent (*p* < 0.05, two‐way repeated‐measures ANOVA on all diets, Figure 2A), as switching from chow to LFD moderated *tau* but not significantly (Figure 2A,C). The average *tau* of these LFD‐fed mice was used to set the *tau*‐like T‐cycle, while the Δ*tau* T‐cycle was set to be shorter than the *tau*‐like T‐cycle by the *tau‐*24‐h deviation (Figure 1A).

Switching from chow to HFD prevented the age‐dependent *tau* changes found under chow and LFD (*p* > 0.05, repeated measure ANOVA for HFD, Figure 2A). Overall, *tau* was longer in HFD‐fed mice than in chow‐fed mice, in general, and longer than in LFD‐fed mice at specific time points (Figure 2A). In parallel, HFD under DD, led to a higher body weight than the average body weight of LFD‐fed mice as from 4 weeks on HFD, marking the onset of diet‐induced obesity (DIO, Figure 3A,B). Defining DIO onset by a more conservative method revealed that HFD resulted in a higher body weight at 6 weeks on the diet (Figure 3A). Compared to LFD, 28 weeks on HFD resulted in a higher fat percentage (i.e., DIO, *p* < 0.001, Figure 3A), due to a higher fat mass and a lower lean mass, as HFD also attenuated the lean mass gain found under LFD (Figure S1A,B). Together, these results show that the HFD‐related longer *tau* preceded, rather than followed, DIO onset (Figures 2A and 3A,B).

### 3.2 Under DD, DIO Onset Is Preceded by Higher Energy Intake and an Early Locomotor Activity Decline

**FIGURE 4:**
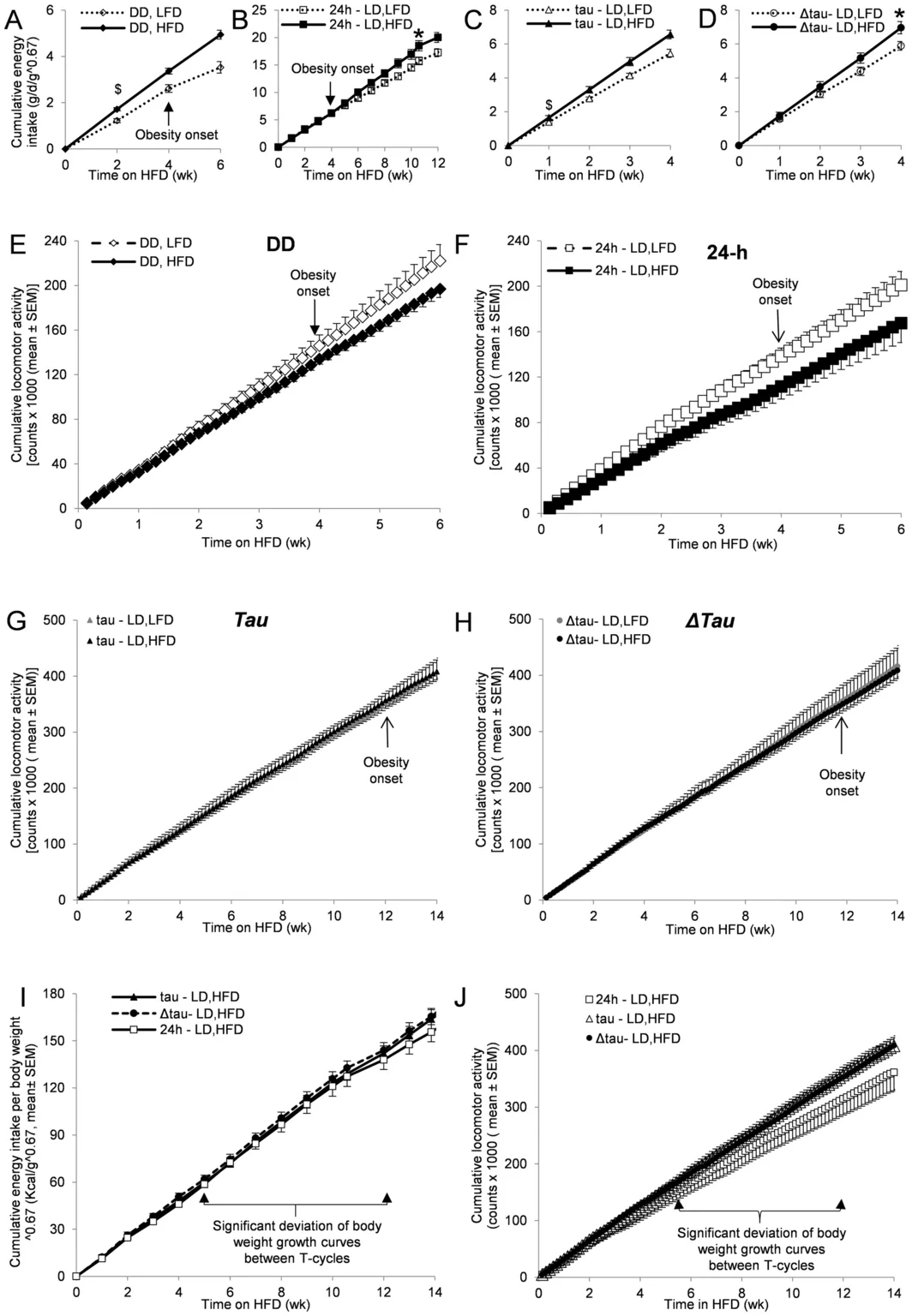
The T‐cycle‐related postponement in high‐fat diet (HFD)‐induced obesity (DIO) onset is accompanied by the prevention of the HFD‐induced reduction in locomotor activity level found under the 24‐h T‐cycle. (A and B) DIO onset under constant darkness (DD) marked at the 4 week on HFD is preceded by a higher cumulative energy intake (corrected for metabolic mass) versus low‐fat diet (LFD)‐fed mice—a phenomenon not found under the 24‐h‐T‐cycle. (C and D) Compared to LFD‐fed mice under *tau* and Δ*tau* regimes, HFD‐fed mice under the same T‐cycle showed a higher cumulative energy intake (corrected for metabolic mass) as early as the first or fourth week on diet, respectively. *p < 0.05, ^$^ *p* < 0.01, significant difference between intakes by Two‐way repeated measure ANOVA with FDR post hoc analysis. (E and F) DIO onset of HFD‐fed mice under DD is followed by reduced cumulative locomotor activity. In contrast, DIO onset under a 24‐h‐T‐cycle is preceded by reduced cumulative activity compared to LFD‐fed mice under the same T‐cycle. (G and H) Under *tau* and Δ*tau* T‐cycles, HFD‐fed mice showed LFD‐resembling locomotor activity levels, before and after DIO onset. (I and H) In HFD‐fed mice, the significant deviation of body weight growth curves between T‐cycles, found between the 6th and 12th week on HFD (see Figure 3G), was not preceded or related to a difference in cumulative energy intake (corrected for metabolic mass, I), but initiated following the trend showing that HFD‐fed mice under 24‐h LD showed reduced cumulative locomotor activity compared to HFD‐fed mice under *tau* and Δ*tau* T‐cycles (J). Data are presented as mean ± SEM, *n* = 8 per group.

HFD did not alter the age‐dependent changes in food intake characterizing LFD‐fed mice (Figure S2A), resulting in a higher cumulative energy intake, even when corrected for metabolic mass (Figures 3C and 4A, and Figure S2B,C). This higher intake was evident as early as the 2nd week on HFD, preceding the DIO onset and the deviation in growth curves at the 4th and 6th week on HFD, respectively, and the gap increased consistently as the HFD‐related higher energy intake was evident throughout most of the experiment (Figures 3A, 4A and Figure S2B,C). Compared to LFD, HFD also resulted in an earlier and steeper decline in locomotor activity (Figure S2D), producing a lower cumulative locomotor activity trend from the 2nd week on HFD, preceding DIO onset, a trend that increased with DIO severity (Figure 4E).

Regression analysis examined the contribution of HFD‐related differences in intake, activity, and *tau* as predictors for the HFD‐related difference in body weight during the first 12 weeks on HFD (Table S2). Cumulative difference in energy intake was positively related to the weight difference (*β* = 0.716, *p* < 0.001), while cumulative difference in locomotor activity was negatively related to the weight difference (*β* = 0.352, *p* < 0.05, Table S2). Notably, the difference in *tau* was on the verge of significance (*p* = 0.07, Table S2). Together, these results indicate that the more positive energy balance of the HFD‐fed mice under DD was related mainly to higher energy intake, but also to lower locomotor activity trend, and, to some extent, to longer *tau* (Table S2).

### 3.3 DIO Onset Is Postponed Under Tau‐Like and Shorter‐Than‐Tau T‐Cycles

Across all T‐cycles, HFD led to DIO, as reflected in a higher fat percentage compared to same‐age LFD‐fed mice (*p* < 0.01, Figure 3D–F). As under DD, DIO was attributed to greater fat mass and lower lean mass, since HFD prevented lean mass gain across all T‐cycles, which did not affect lean mass gain of LFD‐fed mice (Figure S1A,B). Importantly, DIO onset was T‐cycle‐dependent (*p* < 0.001, one‐way ANOVA, Figure 3B,D–F): under the 24‐h T‐cycle, DIO appeared after 4 weeks on HFD (±1.1 week), resembling DD, while *tau* and Δ*tau* T‐cycles postponed DIO onset by 8 weeks to the 12th week on HFD (12.1 ± 1.6 and 11.3 ± 1.6 week, respectively, Figure 3A,B,D–F). This difference was solely related to a divergence in weight curves under HFD (Figure 3G). LFD‐fed mice showed overlapping growth across all T‐cycles (Figure 3H). In contrast, while HFD‐fed mice under *tau* and Δ*tau* T‐cycles showed overlapping growth, their combined curve showed lower body weights versus HFD‐fed mice under the 24‐h T‐cycle (*p* < 0.05, Two‐way repeated measures ANOVA, Figure 3G, insert). This deviation was evident from the first week on HFD and became significant between weeks 7 and 12 on HFD (*p* < 0.05, Figure 3G, insert). Thus, while *tau* and Δ*tau* T‐cycles postpone DIO onset, they do not prevent DIO.

### 3.4 Reduced Locomotor Activity Drives DIO Onset Under a 24‐h T‐Cycle

Food intake across T‐cycles followed the age‐dependent pattern found under DD (Figure S2A,E–G). Compared with LFD, HFD resulted in a lower or similar (varied by T‐cycle) food intake but a higher energy intake due to caloric densities, even after correcting for metabolic mass (Figures 3C and 4A–D and Figures S2 and S3). Under the 24‐h T‐cycle, this cumulative energy gap appeared later than under DD (10th vs. 2nd week on HFD), indicating that increased intake followed, rather than preceded, DIO onset (Figures 3C and 4A,B). In parallel, HFD‐fed mice under the 24‐h T‐cycle showed a lower cumulative locomotor activity trend evident from the 1st week on HFD (Figure 4F). This trend increased with time on HFD and DIO aggravation and resembled the HFD versus LFD trend found under DD (Figure 4E,F and Figure S2H). Regression analysis confirmed that, unlike under DD, the HFD‐LFD weight gap during the first 12 weeks on HFD under 24‐h T‐cycle was mainly related to the HFD‐LFD gap in locomotor activity (*β* = −0.604, *p* < 0.001, Table S3). In parallel, the higher intake remained positively related to the weight difference (*β* = 0.571, *p* < 0.001, Table S3). These findings highlight the centrality of reduced energy expenditure, as evidenced by reduced activity, in the onset of DIO under the 24‐h T‐cycle. At the same time, DIO aggravation is also related to higher energy intake.

### 3.5 The T‐Cycle‐Related Postponement in DIO Onset Is Not Related to Energy Intake Differences

Compared to LFD, HFD under the *tau* and ∆*tau* T‐cycles resulted in a higher cumulative energy intake as early as the 1st or 4th week on HFD, respectively (Figures 3C and 4C,D and Figure S3D,F). However, DIO under *tau* and ∆*tau* T‐cycles was postponed until Week 12 on HFD—about 8–11 weeks later than intake deviations, and about eight weeks later than DIO onset under DD and 24‐h‐T‐cycle (Figure 3B,D,F). Moreover, body weights of HFD‐fed mice under the 24‐h cycle were higher than those under *tau* or ∆tau from the 7th to the 12th week on HFD, despite similar intakes (Figures 3C,G and 4I and Figures S3H and S4F). This suggests that DIO postponement under the *tau* and ∆*tau* reflects a transient increase in energy expenditure that counterbalances elevated intake, as supported, to some extent, by locomotor activity data—see below (Figure 4 and Figures S2 and S5).

### 3.6 Postponement of DIO Onset Is Related to Preservation of Locomotor Activity

Unlike HFD‐fed mice under the 24‐h T‐cycle, mice fed HFD under the *tau* and ∆*tau* T‐cycles did not show reduced activity trend (vs. LFD‐fed mice) preceding DIO, but only long after its onset (Figure 4F–H and Figure S5A–E). In parallel, they maintained a higher cumulative locomotor activity trend than 24‐h HFD‐fed mice from the 2nd week on HFD—preceding the deviation in growth curves between HFD‐fed mice (Figures 3G, insert, and 4J, and Figure S5G). Regression analysis confirmed that T‐cycle (*tau* vs. Δ*tau*) did not affect their weight difference versus 24‐h T‐cycle. In contrast, weight differences of *tau* and Δ*tau* combined versus 24‐h T‐cycle were related to a difference in cumulative activity (*β* = −0.478, *p* < 0.001, Table S4). Together, these results suggest that delayed DIO under *tau* and ∆*tau* T‐cycles, as well as their temporal lower body weight compared to HFD‐fed mice under the 24‐h T‐cycle, are related to higher locomotor activity, indicating a higher energy expenditure.

DIO eventually appeared under the *tau* and ∆*tau* T‐cycles at 12 weeks on HFD, with mice heavier than LFD‐fed controls from the 16th week on diet (Figure 3B,E–F). This postponed DIO onset was not linked to preceding (nor following) attenuation in activity level compared to LFD‐fed mice, but instead followed the consistently increasing cumulative energy intake gap (Figure 4C,D,G,H and Figures S3G,H and S5C,D). By Week 13 on diet, body weight difference among HFD‐fed mice became insignificant (Figure 3G, insert), despite a persistent increment in cumulative activity level favoring HFD‐fed mice under *tau* and ∆*tau* T‐cycles (Figure 4J and Figure S5G). Energy intake, however, showed an emerging trend of a higher energy intake under *tau* and ∆*tau* T‐cycles compared to under 24‐h T‐cycle (Figure 4I and Figure S5F). Together, it is suggested that the postponed DIO reflects maintaining an elevated energy expenditure, whereas the inevitable DIO is explained by the eventual, much higher (HFD‐induced) cumulative energy intake. Notably, the postponed onset of DIO under the *tau* and ∆*tau* T‐cycles appeared at about the same time that the gap between age‐predicted *tau* under LFD versus HFD was at its peak (Figure 2A).

### 3.7 Housing Mice Under the *Tau*‐Like T‐Cycle Does Not Fully Mimic Free‐Running (DD) Conditions

Compared with DD, the *tau*‐like T‐cycle did not affect the growth curve, body weight and composition, and food and energy intake of LFD‐fed (Figures 3C,H and 4 and Figures S1B, S4G, S5F). Additionally, although the *tau*‐like T‐cycle postponed the onset of DIO in HFD‐fed mice relative to DD, it did not prevent DIO or alter final body weight and composition (Figure 3G and Figure S1A). Locomotor activity in LFD‐fed mice was, however, affected by the *tau*‐like T‐cycle, as it resulted in a lower locomotor activity level compared to DD (Figure S5H). The *tau*‐like T‐cycle also interfered with the effect of HFD on locomotor activity, since it was not accompanied by the HFD‐induced reduction in activity levels found under DD (Figure 4E vs. G, and Figure S5C vs. H), resulting in the elimination of the *tau*‐DD difference in activity levels seen in LFD‐fed mice (Figure S5H). In conclusion, keeping mice under a *tau*‐like T‐cycle yields phenotypes similar to those obtained under DD, excluding parameters directly (or indirectly, through diet) related to locomotor activity levels.

### 3.8 In Mature Mice, the *Tau*‐Like T‐Cycle Attenuates DIO Aggravation Through a Higher‐Than‐Expected Energy Expenditure Trend

**FIGURE 5:**
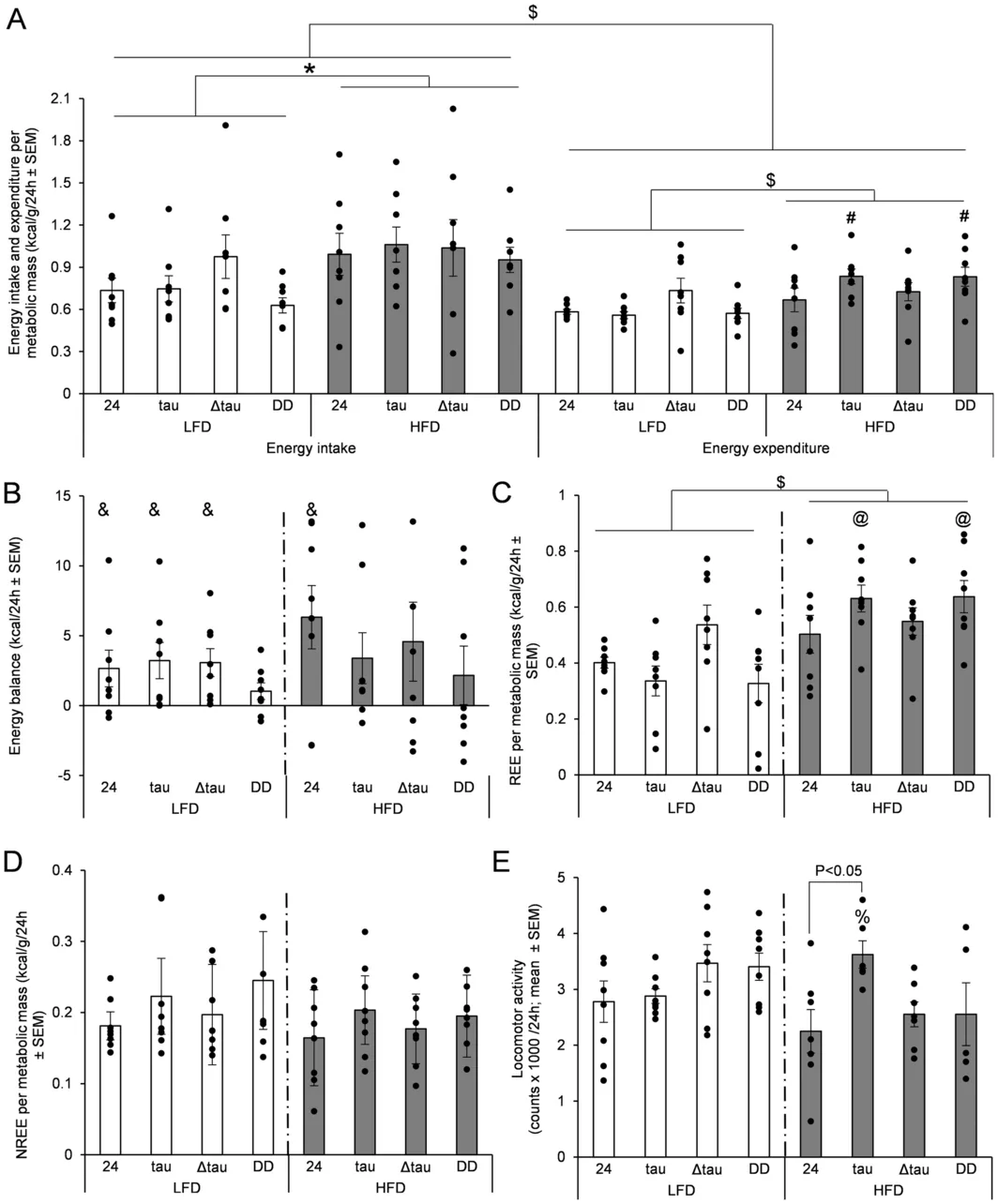
The reduced DIO aggravation under the *tau* T‐cycle in mature mice is associated with a higher‐than‐expected energy expenditure trend measured by indirect calorimetry at 35‐weeks of age. (A) Metabolically mass‐corrected total 24‐h energy intake and expenditure rates were not affected by T‐cycle (regardless of diet), were higher in high‐fat diet (HFD)‐fed mice than in low‐fat diet (LFD)‐fed mice, particularly under *tau* T‐cycle and DD (constant darkness), and intakes were higher than expenditures (*p < 0.05, ^$^ *p* < 0.001, by Two or Three‐way ANOVA). (B) Hence, energy balance (i.e., total 24‐h energy intake minus expenditure, all metabolic‐mass corrected) was significantly positive in LFD‐fed mice under 24‐h, *tau*, and ∆*tau* T‐cycles, and in HFD‐fed mice under 24 T‐cycle (^&^ *p* < 0.05 vs. zero by Wilcoxon test). (C and D) The HFD‐induced elevation in total 24‐h energy expenditure was explained mainly by a trend of a higher resting energy expenditure (REE), particularly under *tau* and DD, but also by the same trend in non‐resting energy expenditure (NREE) (^$^ *p* < 0.001, by Two‐way ANOVA; ^@^ *p* < 0.05 vs. LFD by FDR post hoc analysis). (E) Locomotor activity rates, measured over the two weeks following the indirect calorimetry measurements, followed and explained, to some extent, the T‐cycle and dietary‐related energy expenditure trends (%, *p* < 0.05 vs. LFD, by FDR post hoc analysis). The data are presented as mean ± SEM, *n* = 8 per group.

To account for body mass and composition differences during calorimetry measurements (Figure 3), energy intake and expenditure of mature mice were corrected to lean mass (see Section 2). HFD‐fed mice consumed and expended more calories than LFD‐fed mice (*p* < 0.05 and *p* < 0.001, respectively, Figure 5A). Within diets, T‐cycle did not affect intakes or expenditures, which were lower than intakes (*p* < 0.001, Figure 5A). Thus, LFD‐fed mice showed a positive energy balance under all T‐cycles (*p* < 0.05, Figure 5B), reflected in the continuous weight gain during the calorimetry measurements (Figure 3H). Under HFD, however, positive energy balance was found only under the 24‐h T‐cycle (*p* < 0.05, Figure 5B). This was not due to a higher energy intake compared to other HFD‐fed mice, but due to a lower energy expenditure trend, which was the lowest among HFD‐fed mice and similar to LFD‐fed mice under 24‐h T‐cycle (Figure 5A). By contrast, HFD‐fed mice under the *tau*‐T‐cycle had the highest expenditure, counterbalancing their elevated energy intake and significantly exceeding that of LFD‐fed mice (Figure 5A). These differences in total energy expenditure were reflected in both resting and non‐resting energy expenditure trends (Figure 5C,D). These trends align with the lower locomotor activity in 24‐h HFD‐fed mice compared to *tau*‐HFD‐fed mice, measured during the two weeks following calorimetry measurements (Figure 5E).

### 3.9 A Shorter‐Than‐*Tau* T‐Cycle Results in a Larger Aftereffect Than a Longer‐Than‐*Tau* T‐Cycle

**FIGURE 6:**
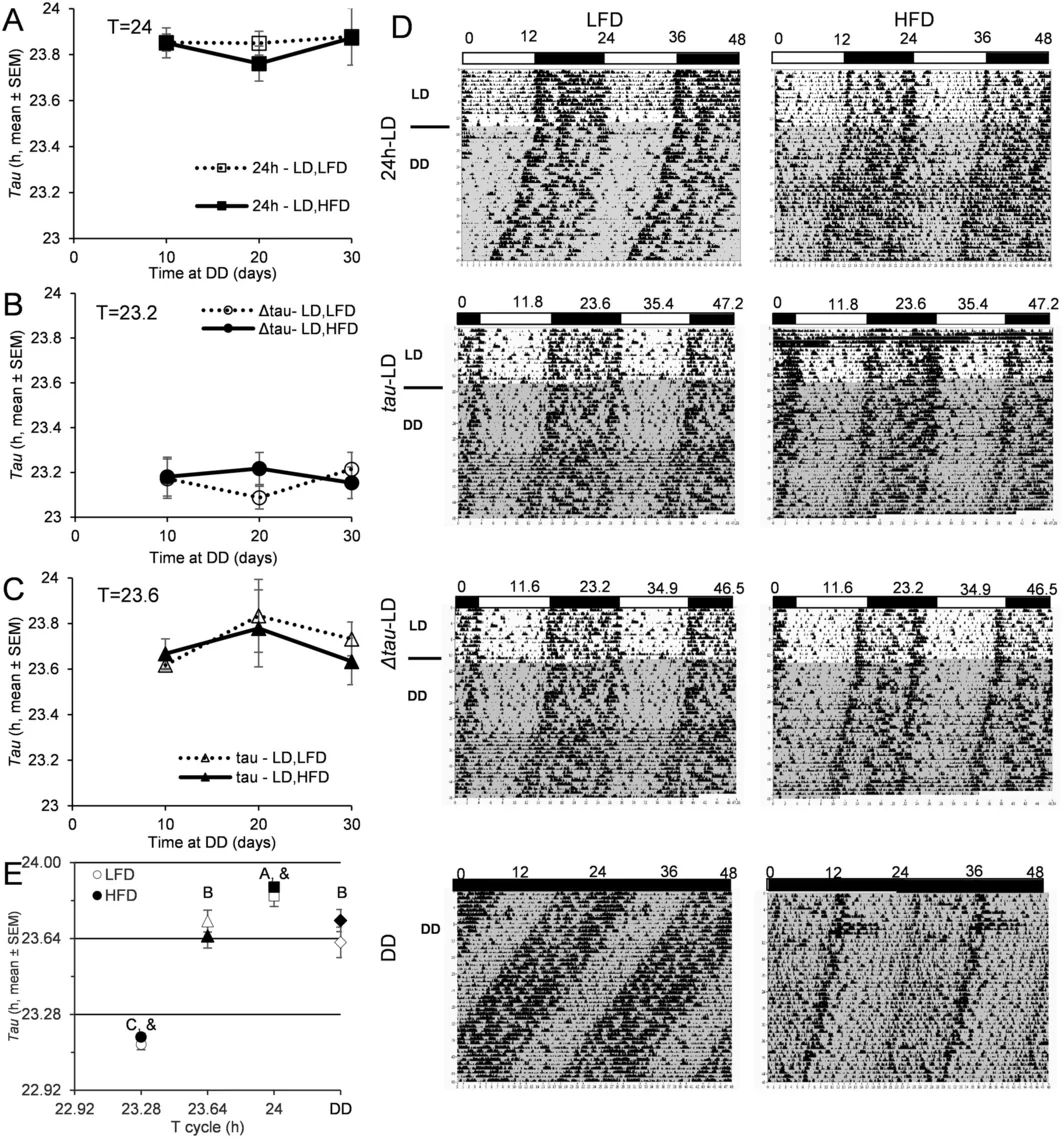
Housing mice under non‐*tau*‐like T‐cycles showed an “aftereffect”. That is an effect on *tau* length; yet the effect was more prominent following a shorter‐than‐*tau* than under a longer‐than‐*tau* T‐cycle. (A–D) High‐fat diet (HFD)‐fed obese and low‐fat diet (LFD)‐fed lean mice transferred from a light–dark photic cycle (LD) to constant darkness (DD) for the first time at 61 weeks of age showed similar *tau*s, which were not affected by the time under DD, regardless of the preceding T‐cycle length. (E) *Tau* length following the *tau*‐like T‐cycle (23.64 h) resembled that of mice continually housed under DD, regardless of diet, and did not differ from the preceding T‐cycle period length. *Tau* length following the 23.28‐h T‐cycle was shorter than the age‐expected *tau* found under DD and the period length of the preceding T‐cycle (23.28‐h). The *tau* lengths following the 24‐h T‐cycle were, however, shorter rather than longer than the preceding T‐cycle period length (i.e., 24‐h) yet were still longer than that under DD or *tau*‐T‐cycle. The data are presented as mean ± SEM, *n* = 5–8 per group. Different letters represent significantly different *tau* (*p* < 0.05) by FDR post hoc analysis following two‐way ANOVA. ^&^ *p* < 0.05 versus period length of the previous T‐cycle period length, by the Wilcoxon test.

After 54 weeks on a diet, mice housed under each one of the three T‐cycles were transferred to DD for *tau* measurement (Figures 1 and 6). Unlike young LFD‐fed mice released from a 24‐h T‐cycle to DD at 5 weeks of age, LFD‐fed mature mice (of 61 weeks of age) showed no age‐related *tau* changes, and HFD (which was associated with long‐term DIO) did not affect *tau* length, as also found in age‐matched DIO mice continually housed under DD (Figures 2A and 6A–D). Thus, the HFD‐related longer *tau* characterizing young mice seems to be restricted to early DIO or young mice, which show a transient shortening in their *tau* under LFD (Figure 2A). Using the *tau* calculated for the whole 30 days under DD showed that *tau* of mature mice was affected, however, by the preceding T‐cycle period length in a dose‐dependent manner: mice housed at a longer‐than‐*tau* T‐cycle showed a longer *tau*, and vice‐versa (i.e., an aftereffect, *p* < 0.001, Figure 6E). In parallel, the *tau*‐like T‐cycle (23.64 h) produced *tau*s indistinguishable from T‐cycle itself and from *taus* of age‐matched mice continually housed under DD and were unaffected by diet (Figure 6E). These results suggest that a T‐cycle oscillating at the age‐expected *tau*‐like period length does not result in an aftereffect.

Notably, shortening the T‐cycle to 23.28‐h (using the 0.36‐h deviation of the LFD‐fed *tau* from 24‐h) resulted in a robust aftereffect: *tau*s of 23.1 ± 0.1‐h (LFD) and 23.2 ± 0.02‐h (HFD), which did not differ by diet, were shorter than the DD‐expected *tau*s, and even shorter than the preceding T‐cycle length (*p* < 0.001, Figure 6E). By contrast, although T‐cycle lengthening by the same magnitude of 0.36‐h (to 24‐h) resulted in *tau*s longer than under DD, the difference versus DD was less than half of the aftereffect found under the ∆*tau* T‐cycle (0.2 ± 0.02‐h vs. 0.5 ± 0.03‐h, Figure 6E). This modest aftereffect resulted in intermediate *tau*s (23.84 ± 0.03‐h, LFD; 23.88 ± 0.02‐h, HFD) that were shorter than the preceding 24‐h T‐cycle period length (*p* < 0.001, Figure 6E). Together, these results show that long‐term housing under non‐*tau*‐like T‐cycles resets *tau* length; and that the directionality of the aftereffect follows the *tau*‐T‐cycle deviation yet is not affected by HFD and the accompanied DIO. Importantly, the aftereffect intensity depends on *tau*‐T‐cycle deviation directionality: a shorter‐than‐*tau* T‐cycle results in a larger aftereffect than a longer‐than‐*tau* T‐cycle.

## 4 Discussion

**FIGURE 7:**
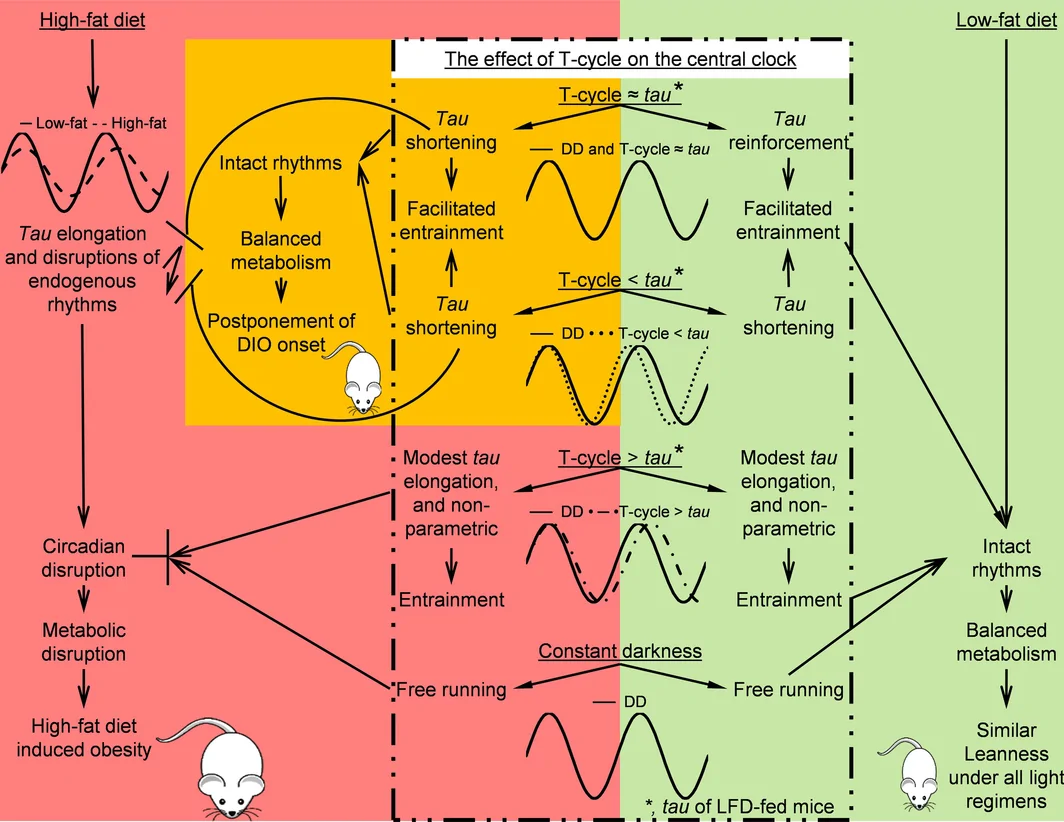
A suggested model for the postponement of high‐fat diet (HFD)‐induced obesity under the *tau*‐like and shorter‐than‐*tau* T‐cycles. DD, constant darkness.

Disruptions of endogenous circadian rhythms due to HFD feeding or chronic misalignment with environmental zeitgebers are established risk factors and mechanistic explanations for metabolic syndrome [6, 33]. This study assessed our “double‐hit” model for DIO [2], which proposed that circadian disruption and the subsequent DIO under the “regular” 24‐h T‐cycle are also secondary to the burden of entrainment to a T‐cycle diverting from *tau* by as little as 0.3 h. Hence, in theory, both can be eliminated under a *tau*‐matching T‐cycle [2]. Our results supported this model: compared to the longer‐than‐*tau* 24‐h T‐cycle, DIO onset was postponed when female mice were entrained to, and hence free‐run, at a period matching the *tau* of age‐matched LFD‐fed mice (23.6 h). Unexpectedly, a shorter‐than‐*tau* T‐cycle (23.4 h) also postponed rather than advanced DIO and exhibited a robust after‐effect. Under DD, which entails no misalignment between endogenous and exogenous cycles, DIO onset resembled that under the 24‐h T‐cycle rather than being postponed. We therefore refined the model to incorporate T‐cycle‐related aftereffects, suggesting that DIO under DD and 24‐h T‐cycle in young female mice is indeed also secondary to an HFD‐induced, age‐related, *tau* lengthening (which was further lengthened by the aftereffect), coupled with reduced energy expenditure under 24‐h LD. According to this refined model, if HFD‐induced *tau* lengthening can be attenuated, DIO onset can be postponed even with ad‐lib HFD feeding, without requiring time‐restricted feeding, underlined by sustaining a higher energy expenditure (Figure 7).

### 4.1 DIO Onset Is Subsequent to HFD‐Induced *Tau* Elongation, Which Is Transient and Unrelated to Long‐Term DIO Severity

Seven‐week‐old female mice fed chow or LFD under DD showed age‐related *tau* shortening, followed by lengthening and stabilization by ca. 33 weeks of age. In contrast, 61‐week‐old LFD‐fed mice released to DD after various T‐cycles showed stable *taus*, indicating that age‐related *tau* changes in young mice reflect maturation rather than time under DD. This age‐related *tau* lengthening aligns with prior findings in non‐HFD‐fed males released to DD at various ages [20, 34]. HFD feeding at 7 weeks of age resulted in a higher body weight, confirming DIO in females, and in longer *taus*, as found in males [21, 35]. *Tau* deviation preceded DIO onset, as observed in males, suggesting that HFD‐driven circadian disruption also predisposes females to DIO [6, 35] (Figure 7). However, our unique long‐term *tau* tracking revealed that the *tau* differences diminished with age due to *tau* lengthening in LFD‐fed mice. This lack of difference was confirmed in mid‐age mice released to DD from three different T‐cycles and aligns with others' and our studies using long‐term‐HFD‐fed mature males [8] and females [2]. Importantly, while weight differences were close to being related to *tau* differences at DIO initiation (*p* = 0.07), weight differences increased with age, even as *tau* differences were stable or vanished. Embedding these results into our model suggests that HFD at a young age induces a slower *tau*, followed by DIO at an initial rate (Figure 7). However, this *tau* deviation is transient and is neither a prerequisite nor related to long‐term DIO severity.

### 4.2 Housing LFD‐Fed Under Near‐*Tau* T‐Cycles Does Not Alter Their Energy Homeostasis

In LFD‐fed mice, entrainment to an age‐corrected *tau*‐like T‐cycle, or to T‐cycles diverted by ±0.4 h from *tau*, did not affect body weight and composition, as well as energy intake and expenditure rates, matching the results under DD. We did observe, however, a higher locomotor activity under DD compared to the *tau*‐like T‐cycle; this likely reflects the removal of light's negative masking effect [36], that is, the light‐induced suppression of activity, activity otherwise shown in the subjective daytime of mice housed under dim light [37, 38], and during the first day under DD [39, 40]. As energy homeostasis under all three T‐cycles matched that under DD, these results suggest that the energy homeostasis of LFD‐fed mice is neither tightened, due to the *tau*‐like T‐cycle that may strengthen the circadian clock, nor loosened, due to the ±0.4‐h clock‐T‐cycle desynchronization. These findings are consistent with our and others' studies for LFD‐fed mice reporting no metabolic impact of entrainable *tau*‐T‐cycle division (from a 2.5‐h faster to a 4‐h slower‐than‐*tau* T‐cycle) [2, 16, 41], while larger non‐entrainable mismatches have been shown to increase weight gain [42, 43]. Our novelty lies in the fact that we are the first to correct the *tau*‐like T‐cycle to age‐related *tau* changes, and to compare the *tau*‐like T‐cycle to DD, as well as to a faster‐than‐*tau* T‐cycle, which is relevant to humans whose *tau* is above 24‐h. Taken together, we suggest the *tau*‐like photic cycle does not over‐strengthen energy balance, and the energy balance of LFD‐fed mice is resistant to the plausible physiological cost of entrainment to entrainable *tau*‐mismatching photic cycles (Figure 7).

### 4.3 Aftereffects Facilitate Entrainment to Near‐*Tau* T‐Cycles

Our long‐term experiments showed that the *tau* of 61‐week‐old LFD‐fed mice shifted toward the duration of the T‐cycle under which they were housed for the preceding 56 weeks, that is, lengthening after exposure to a longer‐than‐*tau* T‐cycle and shortening after a shorter‐than‐*tau* T‐cycle. These results confirm the presence of an “aftereffect” [5], and that by aligning *tau* with the imposed T‐cycle, aftereffects function as a parametric entrainment mechanism [5], thereby doubling entrainment accuracy [44]. The observed *taus* were stable, at least for the 30 days evaluated, as found in some but not all studies [45, 46], supporting the idea that long‐term alterations in the environmental photic cycle can permanently reprogram the endogenous circadian clock [46]. Our novelty is that we are the first to house mice under an age‐corrected *tau*‐matching T‐cycle and to compare a slower versus a faster T‐cycle that are similarly and minimally deviated (by 0.3–0.4 h) from the age‐expected *tau*. This is in contrast to previous studies that compared T‐cycle differing by 1–4 h from an expected (rather than measured) *tau* [45, 46, 47, 48, 49], and were conducted on males only (but see [48]). Our unique *tau*‐matching T‐cycle was not followed by an aftereffect, as predicted by the parametric model of entrainment [50]. Notably, the intensity of our aftereffects depended on the directionality of the symmetrical *tau*‐T‐cycle deviation: the shorter‐than‐*tau* T‐cycle strongly shortened *tau*, whereas the longer‐than‐*tau* 24‐h T‐cycle produced an intermediate *tau*. The results of our unique symmetry study suggest that the parametric entrainment mechanism is sufficient to align the endogenous clock to a photic cycle that is slightly shorter than *tau*. In contrast, a photic cycle similarly longer than *tau* requires additional non‐parametric adjustments, at least for female mice. As stated above, these T‐cycle‐related differences did not alter body weight under LFD, indicating that the entrainment mode does not disturb the energy balance of LFD‐fed mice when the *tau*‐T‐cycle deviation is minimal. This conclusion is embedded in our updated model for energy balance under different diets and lighting regimes (Figure 7).

### 4.4 Entrainment to the 24‐h T‐Cycle Does Not Aggravate DIO Beyond DD, but Is Masking‐Mediated

Under the 24‐h T‐cycle, 7‐week‐old female mice on HFD showed increased body weight due to increased fat mass (i.e., DIO), as found for males [32]. Notably, the growth curve and DIO onset under DD and 24‐h T‐cycle overlapped, indicating that entrainment to a slightly slower‐than‐*tau* T‐cycle (0.3–0.4 h vs. *tau* of HFD or LFD‐fed mice, respectively) does not further disrupt energy homeostasis in young mice beyond that caused by HFD under free‐running conditions. By mid‐age (~40 weeks of age), however, HFD‐fed mice under the 24‐h T‐cycle, but not under DD, showed a positive energy balance, attributed mainly to lower energy expenditure. Interestingly, the mechanisms leading to DIO at a young age differed between DD and the 24‐h T‐cycle. Under DD, DIO was primarily associated with higher energy intake, with only a slight trend toward reduced activity. In contrast, DIO under the 24‐h T‐cycle was preceded by reduced locomotor activity with no change in energy intake. This result is in line with the HFD‐induced immediate activity suppression (along with reduced energy expenditure) seen in HFD‐fed males [21, 51, 52, 53] and in DIO‐prone females [2]. Hence, we suggest that the HFD‐induced reduction in activity under the 24‐h T‐cycle is also light‐mediated via negative masking, as found in prior male studies in which HFD shifted intake to the light phase, yet did not proportionately increase diurnal activity [21, 54]. To sum, following these and our prior [2] and current female studies, we suggest that the immediate HFD‐induced activity reduction under a 24‐h T‐cycle is masking‐mediated, rather than an unavoidable consequence of the HFD, and is not a prerequisite for DIO.

### 4.5 Entrainment to *Tau*‐Like or Faster‐Than‐*Tau* T‐Cycles Similarly Delays DIO Onset, With Delayed Declines in Locomotor Activity Levels

As hypothesized, aligning the photic cycle to the age‐corrected *tau* of LFD‐fed mice (ca. 23.6 h) delayed DIO onset by 8 weeks (to Week 12 on HFD) compared to the 24‐h T‐cycle. Unexpectedly, HFD‐fed mice housed under the even shorter T‐cycle (of ca. 23.4 h, ∆*tau*) exhibited an identical 8‐week delay, rather than the anticipated earlier DIO onset. Thus, both the *tau*‐like and the ∆*tau* T‐cycles, which were respectively shorter by ca. 0.1 and ca. 0.4–0.6 h than the *tau* of HFD‐fed mice, were equally effective in delaying DIO onset. These findings suggest that, within the tested *tau*‐T‐cycle deviation range, DIO postponement is the result of a T‐cycle that is shorter than the *tau* of HFD‐fed mice rather than being related to aligning the photic length with the *tau* of LFD‐fed mice.

Mechanistically, these DIO postponements were not due to reduced energy intake. Instead, both T‐cycles delayed the early decline in locomotor activity typically observed in HFD‐fed mice housed under the 24‐h T‐cycle. In other words, HFD‐fed mice under the *tau*‐like and Δ*tau* T‐cycles sustained higher activity (and likely higher energy expenditure) during the period when 24‐h–entrained HFD‐fed mice become sedentary (with reduced energy expenditure) and obese. Eventually, HFD‐fed mice under both *tau*‐like and Δ*tau* T‐cycles exhibited a trend toward higher cumulative energy and developed DIO, even before activity declined. In summary, we conclude that energy homeostasis under HFD is more tightly regulated when the photic cycle is slightly shorter than the animal's *tau* under HFD, at least in young female mice. Moreover, the similar magnitude of DIO delay observed under *tau*‐like versus Δ*tau* T‐cycles suggests that within 0.1–0.6 deviation from *tau*, the direction of the mismatch (shorter vs. longer) is more influential than the extent of the deviation in determining DIO onset timing (see model at Figure 7).

### 4.6 Aftereffects Offer a Mechanistic Model for DIO Onset Variation

Our findings reveal that HFD and the accompanying DIO did not alter the *tau* observed in DIO‐mature mice when they were released to DD, regardless of prior T‐cycle exposure. Consistent with our findings for LFD‐fed mice, HFD‐fed mice housed under a *tau*‐like T‐cycle exhibited no aftereffect, whereas the shorter‐ and longer‐than‐*tau* T‐cycles induced robust shortening or modest lengthening of *tau*, respectively. This directional asymmetry suggests that parametric entrainment is sufficient in DIO mice to align the endogenous clock to a photic cycle that is slightly shorter than *tau*. In contrast, longer‐than‐*tau* photic cycles require additional non‐parametric adjustments.

Assuming these aftereffects occur in young mice, as suggested by others' work [45, 46], our findings can refine the current model for DIO by integrating photic cycle‐induced aftereffects as a mechanistic explanation for the observed variation in DIO onset (Figure 7). Consistent with prior studies [6, 7], we propose that ad libitum HFD feeding disrupts endogenous circadian rhythms preceding DIO onset, as reflected in our research by the longer *tau*. Therefore, we suggest that these *tau* changes may not merely correlate with but also contribute to the timing of DIO onset, indicating that DIO is, to some extent, secondary to the HFD‐induced *tau* lengthening at an early age. Consequently, we suggest that preventing the HFD‐induced *tau* elongation, via *tau*‐shortening aftereffects induced by a shorter T‐cycle, can delay DIO onset even without caloric restriction (Figure 7). This concept complements existing models attributing DIO to HFD‐driven reductions in rhythm amplitude and phase coherence [6, 7]—disruptions shown to be mitigated by time‐restricted feeding (TRF) regimes (even without caloric restriction) that reinforce endogenous rhythms and attenuate DIO [7, 8] (Figure 7). From our perspective, *tau* shortening manipulation could synergize with TRF regimes in maintaining metabolic homeostasis under obesogenic conditions.

### 4.7 Model Limitations and Open Questions

Our suggested model still leaves open questions, especially regarding the mechanism that delays rather than prevents or attenuates DIO. The similar timing of DIO onset under both *tau*‐like and Δ*tau* T‐cycles, and mainly that it occurred despite reaching the maximal HFD‐induced *tau* differences (i.e., the largest aftereffect), suggests that the timing reflects an HFD‐induced time‐dependent process that eventually overrides the suggested T‐cycle effects. Hence, we propose that while shorter T‐cycles overcome the HFD‐induced *tau* lengthening, thereby postponing DIO onset, they fail to counteract the HFD‐induced desynchronization and damping of biochemical rhythms, which eventually outweigh the protective effect of the T‐cycle, resulting in the inevitable emergence of DIO. This hypothesis will be evaluated in future studies. Our study has limitations, mainly that it examined only female mice, a population underrepresented in DIO research despite the higher prevalence of obesity among women compared to men [23]. Future research should include male mice, extend the findings to other metabolic syndrome traits (e.g., diabetes, insulin resistance), and determine whether T‐cycle‐induced aftereffects precede DIO onset postponement. These studies should also assess whether *tau*‐like and Δ*tau* T‐cycles mitigate the biochemical circadian disruptions induced by HFD—an effect already demonstrated for TRF [7, 8, 25].

### 4.8 Implications for Humans

Although this experimental manipulation has not been and probably cannot be evaluated in humans, an illustrative extrapolation to humans would yield a 5.5‐year delay in DIO, given an average lifespan of 2.2 years for B6 female [55] mice and 80.2 years for US women [56]. We emphasize that this calculation is purely hypothetical and meant only to illustrate scale, as numerous genetic, environmental, and lifestyle factors make human obesity far more complex. Our findings, however, offer mechanistic insights relevant to human chronobiology. Epidemiological studies indicate that individuals with a shorter *tau*, which is typically associated with an early (lark) chronotype [57], exhibit a lower risk of obesity compared to those with a late (owl) chronotype, independent of sleep duration, energy expenditure (activity levels), and other factors [58]. These observations support the “resonance hypothesis,” which argues that minimizing the gap between human *tau* (of ca. 24.5–25 h) and the environmental clock of about 24‐h may mitigate DIO [12]. However, it is also possible to interpret these results in accordance with our hypothesis, which argues that a fast clock counteracts the HFD‐induced *tau* lengthening, thereby postponing (or attenuating) the HFD‐dependent disturbance in biochemical rhythms that leads to DIO. To sum up, a slightly faster clock appears to be more health‐protective than a slower clock, at least under a HFD.

## 5 Conclusion

Together, our findings identify HFD‐induced *tau* lengthening and the accompanying early decline in energy expenditure (reflected by declining locomotor activity) as key determinants of DIO timing in young female mice housed under the conventional 24‐h LD cycle. Accordingly, modestly shortening the photic cycle to be slightly shorter than the HFD‐lengthened *tau*, which resulted in a *tau*‐shortening aftereffect, postponed both the HFD‐associated decline in activity and DIO onset (Figure 7). Hence, it is suggested that early DIO onset is, at least in part, secondary to HFD‐induced *tau* lengthening. Although the tested photic manipulations are not translatable to humans, our results motivate screening for nutritional, behavioral, or pharmacological interventions that modestly shorten *tau* while preserving stable entrainment to the 24‐h cycle, followed by evaluating their capacity to delay or attenuate DIO under ad lib HFD feeding. Finally, as both diet‐ and non‐diet‐induced obesity appear secondary to circadian disruption, we propose the term “circadian disruption–induced obesity” (CDIO) to precisely describe the underlying mechanism.

## Author Contributions

Anat Neumann performed the research. Anat Neumann and Roee Gutman analyzed the data and authored the paper.

## Funding

The research leading to the presented results was funded by the German‐Israel Foundation for Scientific Research and Development, agreement no. I‐2337‐203.13/2014, the European Union's Seventh Framework Programme FP7‐REGPOT‐2012‐2013‐1, agreement no. 316157, and the Israel Science Foundation, agreement no. 2442/21.

## Conflicts of Interest

The authors declare no conflicts of interest.

## Supporting information


**Table S1:** Defining the high‐fat diet‐induced obesity onset at the population level using the mouse‐specific diet‐induced obesity onset.
**Table S2:** Multiple regression of body weight difference between high‐fat diet (HFD) and low‐fat diet (LFD)‐fed mice up to the 12th week on a diet under constant darkness, on delta (HFD minus LFD) cumulative 24‐h energy intake per body weight^0.67^, delta cumulative 24‐h activity, and delta *tau* (the period length of the locomotor activity rhythm under constant darkness conditions).
**Table S3:** Multiple regression of body weight difference between HFD and LFD‐fed mice up to 12th week on a diet under 24 h LD, on delta (HFD minus LFD) cumulative 24‐h energy intake per body weight^0.67^, and delta cumulative 24‐h activity.

**Figure S1:** The effect of high‐fat diet (HFD) on body weight and body composition under constant darkness (DD), and 24‐h, *tau*, and ∆tau T‐cycles measured at the experimental endpoint (before calorimetry measurement) by NMR.
**Figure S2:** The effect of high‐fat diet (HFD) on total 24‐h food and energy intake (also when corrected for metabolic mass), and locomotor activity under constant darkness (DD), on food intake under 24‐h, *tau*, and ∆tau T‐cycles, and on locomotor activity under 24‐h T‐cycle, all compared to low‐fat diet (LFD)‐fed mouse.
**Figure S3:** The effect of a high‐fat diet (HFD) on energy intake under 24‐h, *tau*, and ∆*tau* T‐cycles, compared to a low‐fat diet (LFD).
**Figure S4:** The effect of a high‐fat diet (HFD), compared to a low‐fat diet (LFD), on food intake (A and B), energy intake (C and D), metabolic‐mass corrected energy intake (E and F), and cumulative metabolic‐mass corrected energy intake (G and H) under all photic cycles.
**Figure S5:** The effect of a high‐fat diet (HFD), compared to a low‐fat diet (LFD), on locomotor activity under DD, and 24‐h, *tau*, and ∆*tau* T‐cycles.

## Data Availability

The data supporting this study's findings are available on request from the corresponding author.

## References

[1] R. Steckler , A. Shabtay‐Yanai , M. Pinsky , M. Rauch , S. Tamir , and R. Gutman , “Long‐Lived αMUPA Mice Show Reduced Sexual Dimorphism in Lifespan, and in Energy and Circadian Homeostasis‐Related Parameters,” Journals of Gerontology. Series A, Biological Sciences and Medical Sciences 71 (2016): 451–460. 25863036 10.1093/gerona/glv019

[2] R. Steckler , S. Tamir , and R. Gutman , “Mice Held at an Environmental Photic Cycle Oscillating at Their Tau‐Like Period Length Do Not Show the High‐Fat Diet‐Induced Obesity That Develops Under the 24‐Hour Photic Cycle,” Chronobiologia International 38 (2021): 598–612. 10.1080/07420528.2020.1869029 33455455

[3] S. M. Reppert and D. R. Weaver , “Coordination of Circadian Timing in Mammals,” Nature 418 (2002): 935–941. 12198538 10.1038/nature00965

[4] C. S. Pittendrigh and S. Daan , “A Functional Analysis of Circadian Pacemakers in Nocturnal Rodents – IV. Entrainment: Pacemaker as Clock,” Journal of Comparative Physiology. A 106 (1976): 291–331.

[5] C. S. Pittendrigh and S. Daan , “A Functional Analysis of Circadian Pacemakers in Nocturnal Rodents – I. The Stability and Lability of Spontaneous Frequency,” Journal of Comparative Physiology. A 106 (1976): 223–252.

[6] S. Panda , “Circadian Physiology of Metabolism,” Science (1979) 354 (2016): 1008–1015. 10.1126/science.aah4967 PMC7261592 27885007

[7] M. Hatori , C. Vollmers , A. Zarrinpar , et al., “Time‐Restricted Feeding Without Reducing Caloric Intake Prevents Metabolic Diseases in Mice Fed a High‐Fat Diet,” Cell Metabolism 15 (2012): 848–860. 22608008 10.1016/j.cmet.2012.04.019 PMC3491655

[8] H. Sherman , Y. Genzer , R. Cohen , N. Chapnik , Z. Madar , and O. Froy , “Timed High‐Fat Diet Resets Circadian Metabolism and Prevents Obesity,” FASEB Journal 26 (2012): 3493–3502. 22593546 10.1096/fj.12-208868

[9] R. Gutman , Y. Genzer , N. Chapnik , R. Miskin , and O. Froy , “Long‐Lived Mice Exhibit 24h Locomotor Circadian Rhythms at Young and Old Age,” Experimental Gerontology 46 (2011): 606–609. 21376793 10.1016/j.exger.2011.02.015

[10] C. A. Wyse , A. N. Coogan , C. Selman , D. G. Hazlerigg , and J. R. Speakman , “Association Between Mammalian Lifespan and Circadian Free‐Running Period: The Circadian Resonance Hypothesis Revisited,” Biology Letters 6 (2010): 696–698. 20392719 10.1098/rsbl.2010.0152 PMC2936147

[11] S. Libert , M. S. Bonkowski , K. Pointer , S. D. Pletcher , and L. Guarente , “Deviation of Innate Circadian Period From 24h Reduces Longevity in Mice,” Aging Cell 11 (2012): 794–800. 22702406 10.1111/j.1474-9726.2012.00846.x PMC3526942

[12] C. A. Wyse , “Does Human Evolution in Different Latitudes Influence Susceptibility to Obesity via the Circadian Pacemaker?: Migration and Survival of the Fittest in the Modern Age of Lifestyle‐Induced Circadian Desynchrony,” BioEssays 34 (2012): 921–924. 22933057 10.1002/bies.201200067

[13] M. W. Hurd and M. R. Ralph , “The Significance of Circadian Organization for Longevity in the Golden Hamster,” Journal of Biological Rhythms 13 (1998): 430–436. 9783234 10.1177/074873098129000255

[14] M. Oklejewicz and S. Daan , “Enhanced Longevity in Tau Mutant Syrian Hamsters, *Mesocricetus auratus* ,” Journal of Biological Rhythms 17 (2002): 210–216. 12054192 10.1177/07430402017003004

[15] T. A. Martino , G. Y. Oudit , A. M. Herzenberg , et al., “Circadian Rhythm Disorganization Produces Profound Cardiovascular and Renal Disease in Hamsters,” American Journal of Physiology. Regulatory, Integrative and Comparative Physiology 294 (2008): 1257–1259. 10.1152/ajpregu.00829.2007 18272659

[16] L. Zhou , K. C. Summa , C. Olker , M. H. Vitaterna , and F. W. Turek , “Altered Body Weight Regulation in CK1ϵ Null and Tau Mutant Mice on Regular Chow and High Fat Diets,” Genetics Research International 2016 (2016): 4973242. 27144030 10.1155/2016/4973242 PMC4837286

[17] C. S. Pittendrigh and D. H. Minis , “Circadian Systems: Longevity as a Function of Circadian Resonance in *Drosophila melanogaster* ,” Proceedings of the National Academy of Sciences of the United States of America 69 (1972): 1537–1539. 4624759 10.1073/pnas.69.6.1537 PMC426743

[18] R. Gutman , M. Barnea , L. Haviv , N. Chapnik , and O. Froy , “Peroxisome Proliferator‐Activated Receptor Alpha (PPARalpha) Activation Advances Locomotor Activity and Feeding Daily Rhythms in Mice,” International Journal of Obesity (London) 36 (2012): 1131–1134. 10.1038/ijo.2011.215 22064158

[19] C. I. Eastman , V. A. Tomaka , and S. J. Crowley , “Sex and Ancestry Determine the Free‐Running Circadian Period,” Journal of Sleep Research 26 (2017): 547–550. 28332253 10.1111/jsr.12521 PMC5591035

[20] V. S. Valentinuzzi , K. Scarbrough , J. S. Takahashi , and F. W. Turek , “Effects of Aging on the Circadian Rhythm of Wheel‐Running Activity in C57BL/6 Mice,” American Journal of Physiology. Regulatory, Integrative and Comparative Physiology 273 (1997): R1957–R1964. 10.1152/ajpregu.1997.273.6.R1957 9435649

[21] A. Kohsaka , A. D. Laposky , K. M. Ramsey , et al., “High‐Fat Diet Disrupts Behavioral and Molecular Circadian Rhythms in Mice,” Cell Metabolism 6 (2007): 414–421. 17983587 10.1016/j.cmet.2007.09.006

[22] A. K. Beery and I. Zucker , “Sex Bias in Neuroscience and Biomedical Research,” Neuroscience and Biobehavioral Reviews 35 (2011): 565–572. 20620164 10.1016/j.neubiorev.2010.07.002 PMC3008499

[23] I. Zucker and A. K. Beery , “Males Still Dominate Animal Studies,” Nature 465 (2010): 690. 20535186 10.1038/465690a

[24] K. L. Branecky , K. D. Niswender , and J. S. Pendergast , “Disruption of Daily Rhythms by High‐Fat Diet Is Reversible,” PLoS One 10 (2015): e0137970. 26366733 10.1371/journal.pone.0137970 PMC4569368

[25] K. L. Eckel‐Mahan , V. R. Patel , S. De Mateo , et al., “Reprogramming of the Circadian Clock by Nutritional Challenge,” Cell 155 (2013): 1464–1478. 24360271 10.1016/j.cell.2013.11.034 PMC4573395

[26] C. R. White and R. S. Seymour , “Mammalian Basal Metabolic Rate Is Proportional to Body Mass 2/3,” Proceedings of the National Academy of Sciences of the United States of America 100 (2003): 4046–4049. 12637681 10.1073/pnas.0436428100 PMC153045

[27] R. Gutman , D. Yosha , I. Choshniak , and N. Kronfeld‐Schor , “Two Strategies for Coping With Food Shortage in Desert Golden Spiny Mice,” Physiology & Behavior 90 (2007): 95–102. 17045622 10.1016/j.physbeh.2006.08.033

[28] J. Bass , “Circadian Topology of Metabolism,” Nature 491 (2012): 348–356. 23151577 10.1038/nature11704

[29] J. B. d. V. Weir , “New Methods for Calculating Metabolic Rate With Special Reference to Protein Metabolism,” Journal of Physiology 109 (1949): 1–9. 15394301 10.1113/jphysiol.1949.sp004363 PMC1392602

[30] D. Zecharia , M. Rauch , A. Sharabi‐Nov , S. Tamir , and R. Gutman , “Postnatal Administration of Leptin Antagonist Mitigates Susceptibility to Obesity Under High‐Fat Diet in Male αMUPA Mice,” American Journal of Physiology. Endocrinology and Metabolism 317 (2019): E783–E793. 31454257 10.1152/ajpendo.00099.2019

[31] M. H. Tschöp , J. R. Speakman , J. R. S. Arch , et al., “A Guide to Analysis of Mouse Energy Metabolism,” Nature Methods 9 (2012): 57–63. 10.1038/nmeth.1806 PMC3654855 22205519

[32] Y. Ravussin , R. Gutman , S. Diano , et al., “Effects of Chronic Weight Perturbation on Energy Homeostasis and Brain Structure in Mice,” American Journal of Physiology. Regulatory, Integrative and Comparative Physiology 300 (2011): 1352–1362. 10.1152/ajpregu.00429.2010 PMC3119157 21411766

[33] U. Albrecht , “The Circadian Clock, Metabolism and Obesity,” Obesity Reviews 18 (2017): 25–33. 28164453 10.1111/obr.12502

[34] A. R. Mayeda , J. R. Hofstetter , and B. Possidente , “Aging Lengthens TauDD in C57BL/6J, DBA/2J, and Outbred SWR Male Mice ( *Mus musculus* ),” Chronobiology International 14 (1997): 19–23. 9042548 10.3109/07420529709040538

[35] J. Mendoza , P. Pévet , E. Challet , P. Pevet , and E. Challet , “High‐Fat Feeding Alters the Clock Synchronization to Light,” Journal of Physiology 586 (2008): 5901–5910. 18936083 10.1113/jphysiol.2008.159566 PMC2655413

[36] N. Mrosovsky , “Masking: History, Definitions, and Measurement,” Chronobiology International 16 (1999): 415–429. 10442236 10.3109/07420529908998717

[37] S. Ebihara and K. Tsuji , “Entrainment of the Circadian Activity Rhythm to the Light Cycle: Effective Light Intensity for a Zeitgeber in the Retinal Degenerate C3H Mouse and the Normal C57BL Mouse,” Physiology & Behavior 24 (1980): 523–527. 7375573 10.1016/0031-9384(80)90246-2

[38] L. C. E. Steel , S. Tir , S. K. E. Tam , et al., “Effects of Cage Position and Light Transmission on Home Cage Activity and Circadian Entrainment in Mice,” Frontiers in Neuroscience 15 (2022): 832535. 35082600 10.3389/fnins.2021.832535 PMC8784806

[39] R. Refinetti , “Comparison of Light, Food, and Temperature as Environmental Synchronizers of the Circadian Rhythm of Activity in Mice,” Journal of Physiological Sciences 65 (2015): 359–366. 10.1007/s12576-015-0374-7 PMC10717296 25800223

[40] W. J. Schwartz and P. Zimmerman , “Circadian Timekeeping in BALB/c and C57BL/6 Inbred Mouse Strains,” Journal of Neuroscience 10 (1990): 3685–3694. 2230953 10.1523/JNEUROSCI.10-11-03685.1990 PMC6570095

[41] A. Campuzano , T. Cambras , J. Vilaplana , M. M. Canal , M. Carulla , and A. Díez‐Noguera , “Period Length of the Light‐Dark Cycle Influences the Growth Rate and Food Intake in Mice,” Physiology & Behavior 67 (1999): 791–797. 10604852 10.1016/s0031-9384(99)00196-1

[42] I. N. Karatsoreos , S. Bhagat , E. B. Bloss , J. H. Morrison , and B. S. McEwen , “Disruption of Circadian Clocks Has Ramifications for Metabolism, Brain, and Behavior,” Proceedings of the National Academy of Sciences of the United States of America 108 (2011): 1657–1662. 21220317 10.1073/pnas.1018375108 PMC3029753

[43] N. Park , S. Cheon , G. H. Son , S. Cho , and K. Kim , “Chronic Circadian Disturbance by a Shortened Light‐Dark Cycle Increases Mortality,” Neurobiology of Aging 33 (2012): 1122.e11–1122.e22. 10.1016/j.neurobiolaging.2011.11.005 22154820

[44] D. G. Beersma , S. Daan , and R. A. Hut . “Accuracy of Circadian Entrainment Under fluctuating Light Conditions: Contributions of Phase and Period Responses,” Journal of Biological Rhythms 14 (1999): 320–329. 10447313 10.1177/074873099129000740

[45] S. J. Aton , G. D. Block , H. Tei , S. Yamazaki , and E. D. Herzog , “Plasticity of Circadian Behavior and the Suprachiasmatic Nucleus Following Exposure to Non‐24‐Hour Light Cycles,” Journal of Biological Rhythms 19 (2004): 198–207. 15155006 10.1177/0748730404264156

[46] A. Azzi , R. Dallmann , A. Casserly , et al., “Circadian Behavior Is Light‐Reprogrammed by Plastic DNA Methylation,” Nature Neuroscience 17 (2014): 377–382. 24531307 10.1038/nn.3651

[47] P. C. Molyneux , M. K. Dahlgren , and M. E. Harrington , “Circadian Entrainment Aftereffects in Suprachiasmatic Nuclei and Peripheral Tissues In Vitro,” Brain Research 1228 (2008): 127–134. 18598681 10.1016/j.brainres.2008.05.091

[48] J. S. Pendergast , R. C. Friday , and S. Yamazaki , “Photic Entrainment of Period Mutant Mice Is Predicted From Their Phase Response Curves,” Journal of Neuroscience 30 (2010): 12179–12184. 20826680 10.1523/JNEUROSCI.2607-10.2010 PMC2943870

[49] R. E. Mistlberger and M. M. Holmes , “Behavioral Feedback Regulation of Circadian Rhythm Phase Angle in Light‐Dark Entrained Mice,” American Journal of Physiology. Regulatory, Integrative and Comparative Physiology 279 (2000): R813–R821. 10956238 10.1152/ajpregu.2000.279.3.R813

[50] C. H. Johnson , J. A. Elliott , and R. Foster , “Entrainment of Circadian Programs,” Chronobiology International 20 (2003): 741–774. 14535352 10.1081/cbi-120024211

[51] T. L. Moretto , I. D. Benfato , F. P. de Carvalho , M. Barthichoto , L. Le Sueur‐Maluf , and C. A. M. de Oliveira , “The Effects of Calorie‐Matched High‐Fat Diet Consumption on Spontaneous Physical Activity and Development of Obesity,” Life Sciences 179 (2017): 30–36. 28449870 10.1016/j.lfs.2017.04.017

[52] J. Guo and K. D. Hall , “Predicting Changes of Body Weight, Body Fat, Energy Expenditure and Metabolic Fuel Selection in C57BL/6 Mice,” PLoS One 6 (2011): e15961. 21246038 10.1371/journal.pone.0015961 PMC3016341

[53] M. Bjursell , A. K. Gerdin , C. J. Lelliott , et al., “Acutely Reduced Locomotor Activity Is a Major Contributor to Western Diet‐Induced Obesity in Mice,” American Journal of Physiology. Endocrinology and Metabolism 294 (2008): E251–E260. 18029443 10.1152/ajpendo.00401.2007

[54] L. Sun , Y. Wang , Y. Song , et al., “Resveratrol Restores the Circadian Rhythmic Disorder of Lipid Metabolism Induced by High‐Fat Diet in Mice,” Biochemical and Biophysical Research Communications 458 (2015): 86–91. 25640840 10.1016/j.bbrc.2015.01.072

[55] R. Yuan , Q. Meng , J. Nautiyal , et al., “Genetic Coregulation of Age of Female Sexual Maturation and Lifespan Through Circulating IGF1 Among Inbred Mouse Strains,” Proceedings of the National Academy of Sciences of the United States of America 109 (2012): 8224–8229. 22566614 10.1073/pnas.1121113109 PMC3361401

[56] E. Arias , B. Tejada‐Vera , F. Ahmad , and K. D. Kochanek , “Provisional Life Expectancy Estimates for 2020,” (2021).

[57] J. F. Duffy , D. W. Rimmer , and C. A. Czeisler , “Association of Intrinsic Circadian Period With Morningness‐Eveningness, Usual Wake Time, and Circadian Phase,” Behavioral Neuroscience 115 (2001): 895–899. 11508728 10.1037//0735-7044.115.4.895

[58] X. Sun , J. Gustat , S. M. Bertisch , S. Redline , and L. Bazzano , “The Association Between Sleep Chronotype and Obesity Among Black and White Participants of the Bogalusa Heart Study,” Chronobiology International 37 (2020): 123–134. 31747792 10.1080/07420528.2019.1689398 PMC6981036

