# Supplementary material for: Photic Cycle Shorter Than or Equal to Endogenous tau Postpones Diet‐Induced Obesity in Mice and Shows a Robust Aftereffect
Source: FASEB J. 2026 Sep 1;40(17):e72254. doi: 10.1096/fj.202502574RR (PMC13532521; doi:10.1096/fj.202502574RR)
Supplement: Supplementary file 1 [file FSB2-40-e72254-s002.docx]

**Supplementary Tables**

Table S1: Defining the high-fat diet-induced obesity onset at the population level using the mouse-specific diet-induced obesity onset.

| Step | Goal | Data | Statistical test |
| --- | --- | --- | --- |
| 1 | Detecting the effect of a high-fat diet (HFD) on body weight difference vs. a low-fat diet (LFD) | The time on HFD (in weeks) during which the body weight of a HFD-fed mouse was significantly higher than the average body weight of age-matched LFD-fed mice under the same T-cycle. | Wilcoxon |
| 2 | Defining the diet-induced obesity (DIO) onset per mouse. | The minimal time on HFD (in weeks) during which body weight differed significantly (by the Wilcoxon test, see step 1) for two consecutive measurements (Fig. 3. A, D-F). |  |
| 3 | Evaluating the effect of T-cycle on DIO onset. | DIO onset of HFD-fed mice by T-cycle (Fig. 3B). | FDR post hoc analysis following One-way ANOVA |

Table S2: Multiple regression of body weight difference between high-fat diet (HFD) and low-fat diet (LFD)-fed mice up to the 12^th^ wk on a diet under constant darkness, on delta (HFD minus LFD) cumulative 24-h energy intake per body weight^0.67^, delta cumulative 24-h activity, and delta *tau* (the period length of the locomotor activity rhythm under constant darkness conditions).

| Variables | B | SE | β |
| --- | --- | --- | --- |
| Delta cumulative 24-h energy intake per metabolic mass (body weight^0.67^) | 0.110 | 0.026 | 0.716^$^ |
| Delta cumulative 24-h locomotor activity | 2.110E-5 | 0.00 | 0.352^*^ |
| Delta *Tau* | 3.98 | 1.847 | 0.223*^P^*^=0.07^ |
| R^2^ |  | 0.392 |  |
| F |  | 11.183^$^ |  |

LFD, low-fat diet; HFD, high-fat diet; LD, light-dark T-cycle. *, p<0.05; $, p<0.001

Table S3: Multiple regression of body weight difference between HFD and LFD-fed mice up to 12^th^ wk on a diet under 24h LD, on delta (HFD minus LFD) cumulative 24-h energy intake per body weight^0.67^, and delta cumulative 24-h activity.

| Variables | B | SE | β |
| --- | --- | --- | --- |
| Delta cumulative 24-h locomotor activity | -3.045E-5 | 0.00 | -0.604^$^ |
| Delta cumulative 24-h energy intake per metabolic mass (body weight^0.67^) | 0.108 | 0.014 | 0.571^$^ |
| R^2^ |  | 0.477 |  |
| F |  | 46.142^$^ |  |

LFD, low-fat diet; HFD, high-fat diet; LD, light-dark T-cycle. $, p<0.001

Table S4: Multiple regression of body weight difference between HFD-fed mice under *tau* and Δ*tau* T-cycles and 24-h T-cycle (*tau* and Δ*tau* combined minus 24-h), on delta cumulative 24-h locomotor and T-cycle (*tau* vs. Δ*tau*) up to twelve weeks on HFD. The *tau*-LD T-cycle was a symmetrical T-cycle with a period length that was equal to the average *tau* of the same-age mice on a LFD and housed at DD, while the period length of the symmetrical Δ*tau* T-cycles was set to be shorter than the *tau* T-cycle by the age-appropriate deviation of *tau* from 24-h.

| Variables | B | SE | β |
| --- | --- | --- | --- |
| Delta cumulative 24-h locomotor activity | -3.455E-5 | 0.00 | -0.478^$^ |
| T-cycle (*tau* vs. Δ*tau*) | -0.89 | 0.229 | -0.024 |
| R^2^ |  | 0.229 |  |
| F |  | 30.396^$^ |  |

HFD, high-fat diet; $, p<0.001.
