# Supplementary material for: Photic Cycle Shorter Than or Equal to Endogenous tau Postpones Diet‐Induced Obesity in Mice and Shows a Robust Aftereffect
Source: FASEB J. 2026 Sep 1;40(17):e72254. doi: 10.1096/fj.202502574RR (PMC13532521; doi:10.1096/fj.202502574RR)
Supplement: Supplementary file 2 [file FSB2-40-e72254-s001.zip › fsb272254-sup-0002-Supinfo02.docx]

**Supplementary Figure Legends**

**Figure S1**: The effect of high-fat diet (HFD) on body weight and body composition under constant darkness (DD), and 24-h, *tau*, and ∆tau T-cycles measured at the experimental endpoint (before calorimetry measurement) by NMR. *A*) T-cycles did not affect body mass and composition of mice fed a low-fat diet (LFD). Compared to LFD, HFD resulted in similarly higher body weight and fat percentage under all T-cycles. The higher body weight of HFD-fed mice across all T-cycles was due to a higher fat mass that overcompensated for the lower lean mass, with no effect on free fluid mass. *B*) The HFD-related lower lean mass was due to the prevention of the LFD-related lean mass gain, which was found under all T-cycles, but to a lesser extent under DD. The data are presented as mean ± SEM, n = 8 per group. Different letters represent significantly different masses (p < 0.05) as determined by FDR post hoc analysis following a two-way ANOVA. Roman numerals indicate fat mass, lowercase letters indicate muscle mass, uppercase letters indicate body weight, and Greek letters indicate fat percentage.

**Figure S2**: The effect of high-fat diet (HFD) on total 24-h food and energy intake (also when corrected for metabolic mass), and locomotor activity under constant darkness (DD), on food intake under 24-h, *tau*, and ∆tau T-cycles, and on locomotor activity under 24-h T-cycle, all compared to low-fat diet (LFD)-fed mouse. *A*) Under DD, HFD did not affect food intake beyond the age-dependent changes characterizing LFD-fed mice. *B and C*) Therefore, HFD under DD resulted in a higher energy intake (*B*), even when correcting for their metabolic mass (body weight^^0.67^, *C*). *D*) Under DD, HFD resulted in a steeper decrease in activity level than the age-dependent reduction in the activity level characterizing LFD-fed mice. *E – G*) Under the 24-h, *tau,* and ∆*tau* T-cycles, HFD resulted in a transit reduction in food intake compared to LFD-fed mice. *H*) Under the 24-h T-cycle, HFD resulted in a steeper decrease in activity than the age-dependent reduction in the activity level characterizing LFD-fed mice. The data are presented as mean ± SEM, n = 8 per group.

**Figure S3**: The effect of a high-fat diet (HFD) on energy intake under 24-h, *tau*, and ∆*tau* T-cycles, compared to a low-fat diet (LFD). *A – E*) Under 24-h, but mainly under *tau* and ∆*tau* T-cycles, HFD resulted in a higher energy intake (A, C, and E), even when correcting for their metabolic mass (body weight^^0.67^), compared to LFD-fed mice (B, D, and F). *G* and *H*) Compared to LFD, HFD under *tau* and *∆tau* T-cycles resulted in a higher metabolic-mass-corrected cumulative intake from the 2^nd^ and 4^th^ week on HFD (respectively), preceding the HFD-induced obesity onset. *, p<0.05, $, p<0.001, by FDR post-hoc analysis following Two-way repeated measure ANOVA.

**Figure S4:** The effect of a high-fat diet (HFD), compared to a low-fat diet (LFD), on food intake (A and B), energy intake (C and D), metabolic-mass corrected energy intake (E and F), and cumulative metabolic-mass corrected energy intake (G and H) under all photic cycles.

**Figure S5:** The effect of a high-fat diet (HFD), compared to a low-fat diet (LFD), on locomotor activity under DD, and 24-h, *tau*, and ∆*tau* T-cycles. *A – D*) Compared to LFD, HFD resulted in a trend of reduced locomotor activity under *tau* and ∆*tau* T-cycles, yet only after DIO onset. *E*) Under the 24-h T-cycle, however, HFD reduced cumulative locomotor activity preceding DIO onset. *F and G*) Compared to HFD-fed mice under the 24-h T-cycle, HFD-fed mice under the *tau* and ∆*tau* T-cycles showed a trend of a higher cumulative energy intake (corrected for metabolic mass), counterbalanced by a trend of a higher cumulative locomotor activity. At the same time, there was no difference in their body weight. *G*) Compared to HFD under *tau* and ∆*tau* T-cycles, HFD under the 24-h T-cycle resulted in a consistently lower cumulative locomotor activity, preceding and following the deviation in body weight. *H*) Compared to DD, the *tau* T-cycle resulted in a lower cumulative locomotor activity in LFD-fed mice. Compared to LFD, HFD did not affect cumulative locomotor activity under the *tau* T-cycle (see C), while still resulting in a lower cumulative locomotor activity under DD (see lower insert), and it did so to a level that eliminated the T-cycle-related difference found on LFD (see upper insert).
